# Humans use multi-objective control to regulate lateral foot placement when walking

**DOI:** 10.1371/journal.pcbi.1006850

**Published:** 2019-03-06

**Authors:** Jonathan B. Dingwell, Joseph P. Cusumano

**Affiliations:** 1 Department of Kinesiology, Pennsylvania State University, University Park, State College, Pennsylvania, United States of America; 2 Department of Engineering Science & Mechanics, Pennsylvania State University, University Park, State College, Pennsylvania, United States of America; Johns Hopkins University, UNITED STATES

## Abstract

A fundamental question in human motor neuroscience is to determine how the nervous system generates goal-directed movements despite inherent physiological noise and redundancy. Walking exhibits considerable variability and equifinality of task solutions. Existing models of bipedal walking do not yet achieve both continuous dynamic balance control and the equifinality of foot placement humans exhibit. Appropriate computational models are critical to disambiguate the numerous possibilities of how to regulate stepping movements to achieve different walking goals. Here, we extend a theoretical and computational Goal Equivalent Manifold (GEM) framework to generate predictive models, each posing a different experimentally testable hypothesis. These models regulate stepping movements to achieve any of three hypothesized goals, either alone or in combination: maintain lateral *position*, maintain lateral speed or “*heading*”, and/or maintain *step width*. We compared model predictions against human experimental data. Uni-objective control models demonstrated clear redundancy between stepping variables, but could not replicate human stepping dynamics. Most multi-objective control models that balanced maintaining two of the three hypothesized goals also failed to replicate human stepping dynamics. However, multi-objective models that strongly prioritized regulating step width over lateral position did successfully replicate all of the relevant step-to-step dynamics observed in humans. Independent analyses confirmed this control was consistent with linear error correction and replicated step-to-step dynamics of individual foot placements. Thus, the regulation of lateral stepping movements is inherently *multi*-objective and balances task-specific trade-offs between competing task goals. To determine how people walk in their environment requires understanding both walking biomechanics and how the nervous system regulates movements from step-to-step. Analogous to mechanical “templates” of locomotor biomechanics, our models serve as “control templates” for how humans *regulate* stepping movements from each step to the next. These control templates are symbiotic with well-established mechanical templates, providing complimentary insights into walking regulation.

## Author summary

When we walk, we walk in real-world contexts and with specific goal to achieve. Side-to-side movements are paramount because walking bipeds (humans, animals, robots, etc.) are inherently more unstable laterally. This is particularly important in older adults as sideways falls greatly increase hip fracture risk. Additionally, we normally walk on paths that limit (more or less) our lateral movements. Appropriately regulating lateral stepping movements is thus critical to achieving successful locomotion in any such context. Here, we use appropriate models to test competing hypotheses about how humans regulate lateral stepping movements from each step to the next to identify what task goals they try to achieve. Our work both bridges and unifies perspectives from dynamic walking and computational motor control to provide a coherent theoretical and computational framework from which to study motor regulation in the context of goal-directedness across a wide range of walking tasks and/or conditions.

## Introduction

Human movements are subject to both inherent physiological noise [1, 2] and multiple levels of redundancy [3–5]: i.e., the body has more mechanical degrees-of-freedom than needed to execute most movements, more muscles than needed to move a given joint, etc. Likewise, most tasks we perform exhibit equifinality [3, 6–8]: i.e., we can achieve them with equal success by an infinite number of movements [9–12]. It remains a fundamental question in human motor neuroscience to determine how the human nervous system generates accurate and repeatable goal-directed movements in the face of these challenges.

Walking is a highly relevant task that exhibits both considerable variability [13, 14] and equifinality [9, 10, 15]. Lateral (side-to-side) movements in walking are paramount because humans are inherently more unstable laterally [16–18]. This contributes to sideways falls and hip fractures in older adults [19, 20]. External lateral stabilization improves lateral walking stability in young [21] and older [22] adults. Similarly, laterally-directed external perturbations *de*stabilize walking humans far more than comparable anterior-posterior perturbations [23]. To prevent falling, lateral CoM accelerations must be redirected in the opposite direction. The simplest way to achieve this is by appropriate lateral placement of the foot on the subsequent step [17, 24, 25]. However, placing the next foot “lateral to the CoM” still leaves infinite options for where to place that foot [18, 25]. Humans readily adapt foot placements to avoid obstacles and/or to step on specified targets [26–28]. They also actively modulate foot placement to affect lateral maneuvers [29, 30] or to negotiate stabilizing or destabilizing external forces [31]. That humans can readily modulate foot placement across so many contexts strongly suggests a high degree of equifinality in peoples’ choice of where to step.

Experimentally, it has been shown that center of mass state at mid stance strongly predicts subsequent lateral foot placement of that same step [32, 33]. These predictive correlations vary systematically with changes in step width [34] or walking speed [35], decrease in healthy older adults [36], and are disrupted in persons with stroke who have high fall risk [37]. Lateral foot placement is associated with swing phase activity of the primary hip abductor and adductor muscles [32], although the precise contribution of these activations to the observed predictability is not yet clear. However, such correlations by themselves do not necessarily indicate within-step control processes, but could instead arise largely from passive dynamics [25, 33]. Thus, the extent to which these correlations reflect actual “control” remains unresolved.

**Fig 1:**
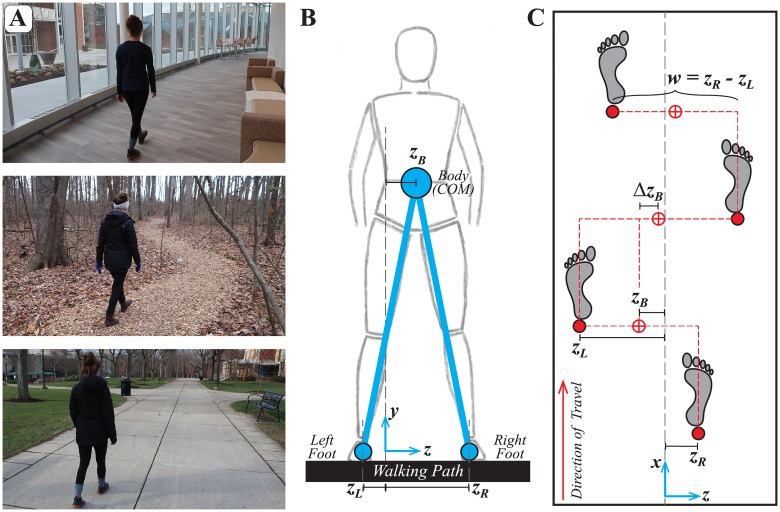
Defining relevant lateral stepping variables. A) Common examples of a person walking on paths with lateral boundaries. Such paths occur both indoors and outdoors, can be wide or narrow, may have borders that are more or less well-defined, etc. B-C) Configuration of bipedal walking during a step as viewed in the (B) frontal and (C) horizontal planes. Coordinates are defined in a Cartesian system with {*x*,*y*,*z*} axes as shown in (B) and (C). For convenience, we set the origin at the geometrical center of the path in the lateral direction. Each diagram shows lateral positions of the feet (*z*_*L*_ and *z*_*R*_) and body (*z*_*B*_; Eq 2—taken here as a proxy for the center-of-mass, CoM). These yield definitions of primary lateral stepping variables (C) that could be regulated from step to step: positions of the individual feet (*z*_*L*_ and *z*_*R*_), lateral body position (*z*_*B*_; Eq 2), change in lateral position (Δ*z*_*B*_; Eq 3), taken here as a proxy for the lateral component of speed or ‘heading’, and step width (*w*; Eq 4).

More importantly for the present study, such empirical correlations demonstrate only how mid-stance body state predicts subsequent foot placement *within a given step* [33] and so cannot address how humans regulate stepping movements *from each step to the next* [9, 10]. However, people walking perform a motor *task* within a context (i.e., an environment; e.g., Fig 1A) and with specific task goals to achieve (e.g., maintain speed, move in a particular direction, stay on a given path, avoid obstacles, etc.). Numerous studies show people use visual information acquired before each step to plan where to place the foot and then execute the step itself as a predominantly ballistic movement [26–28, 38, 39]: i.e., people walk by regulating foot placements across multiple steps. Thus, a complete understanding of walking control requires consistent and complimentary descriptions of both how stepping movements are executed *within* each step [16, 18, 25, 32, 33] and of how those movements are then regulated *between* steps [9, 10, 40]. Appropriate models and analytical methods are therefore needed to generate empirically testable and suitably falsifiable hypotheses about how stepping movements are regulated from each step to the next [9, 10].

Many studies have modeled walking dynamics. Early efforts used inverted pendulum models to show how appropriate foot placement can redirect the center of mass to stabilize walking [41, 42]. Hof derived a ‘Margin of Stability’ [18, 43] that defines the minimum lateral foot placement required to achieve static standing at the end of one step. Koolen et al. [44] derived ‘*N*-step capture regions’ that predict the range of available foot placements a biped can take to achieve static stability after *N* steps. The methods of [18, 44] yield inequality conditions on appropriate foot placement that reflect the inherent equifinality [3, 4, 6] of stepping [45]. However, because the above methods are based on achieving some hypothetical stable standing state, and not the continuous dynamic stability expected in walking, they misestimate actual lateral foot placements in humans [18, 25]. Conversely, Kuo [16] and later Hobbelen et al. [46] proposed lateral foot placement control policies that can maintain continuous dynamic limit cycle motion. However, neither of those proposed controllers considered equifinality in choice of foot placement. Thus, prior studies have developed control strategies that either achieve continuous dynamic balance control [16, 46], or allow for equifinality of foot placement [18, 44] as widely observed in humans [9, 15, 40, 47–50], but not both.

Moreover, when humans walk in the real world, they regularly negotiate a wide variety of environmental contexts [51], both indoors and outdoors [27, 28], perhaps containing fixed [27, 28, 51] and/or moving [52, 53] obstacles to negotiate. In nearly all of these contexts, humans walk on paths that in some way restrict their lateral movements (e.g., Fig 1A). The lateral boundaries of such paths are sometimes well-defined (e.g., building hallways, store aisles, etc.), or sometimes less so (e.g., outdoor walking paths [28]), etc.). Appropriately regulating lateral stepping movements is critical to achieving successful locomotion in these contexts. However, most models of how people navigate environments [52–54] consider the person as a point mass and do not address the question of how people regulate their stepping movements to *achieve* this navigation [55]. Thus, it is possible purposeful (goal-directed) walking requires precise step-to-step control of position and/or heading to stay on one’s desired path (or trajectory) [52, 53]. Conversely, people may adopt less stringent control that corrects errors in position and/or heading only when they become “sufficiently large” as to need correcting [3, 8, 12]. To our knowledge, the question of how humans regulate their lateral stepping movements from each step to the next within their respective environment has not been addressed, either experimentally or computationally.

Here, we adopt a computational framework to generate concrete, experimentally testable *a priori* hypotheses [7, 8] to address these questions. This framework is founded on the idea of a *goal function* that theoretically defines a task and, in the presence of equifinality [3, 4], determines the sets of all possible task solution strategies: i.e., a goal equivalent manifold or GEM [6–8, 12]. We previously successfully implemented this approach to identify how humans regulate stride-to-stride stepping movements in the sagittal plane [9, 10]. Several independent studies replicated those primary findings [48–50] and/or subsequently confirmed other main theoretical predictions experimentally [40, 47, 56]. Analogous to mechanical “templates” of locomotor biomechanics [57–59], our prior models [9, 10] serve as “control templates” for how humans *regulate* sagittal plane stepping movements: i.e., they contain the minimal number of variables and parameters needed to fully capture the relevant walking behavior [57, 59]. Here, we extend this approach to develop goal functions and computational models that pose testable hypotheses for how humans (or other bipeds) might regulate *lateral* stepping movements as they walk on some specified path.

## Results

### Defining the task and relevant variables

We consider the common task of some biped (ostensibly a human, but could more generally be any robot, walking model, etc.) walking on any clearly defined path with identifiable lateral boundaries (e.g., Fig 1A). We treat walking control as hierarchical. We assume some within-step processes (be they active, passive, or both) generate each individual step [16, 18, 25, 32, 33]. We presume those processes will be different for different bipeds and our models are purposefully agnostic to such details. Indeed, a strength of our approach is precisely that it can apply to *any* reasonable biped (human, robot, etc.), regardless of how that biped may be actuated and/or controlled at a mechanical (and/or possibly neuromuscular) level to generate each step [9].

What we study here is the *between*-step regulation of the output of that process. That is, conceptually, we consider the result of each stepping motion as the input to our models [9, 10, 40] of the between-step regulation needed to achieve goal-directed walking [26–28, 38, 39]. We focus on regulating lateral stepping movements in the frontal plane because lateral balance is a significant challenge in bipedal walking [16, 21, 23, 60] and because sideways falls are especially dangerous for elderly adults [19, 20, 61]. We further consider lateral stepping as decoupled from sagittal plane movement (i.e., forward progression). While there is some coupling between frontal and sagittal planes, it is minimal [17, 25, 46, 60] and our prior work has already addressed how humans regulate sagittal plane stepping [9, 10].

The simplest, mechanically sufficient description of the frontal plane configuration of the lateral components of a stepping biped (Fig 1B) includes locations of the body (ostensibly the center-of-mass) and of each of the two feet [33, 46, 62]. Extending our prior sagittal plane work [9, 10], the primary task requirement for any such biped is simply to not step off the path, in this case to either side, i.e.:

$$\forall \;n\in \left\{1,\ldots ,N\right\}:\;-\frac{W_{P}\left(x\right)}{2}<\left\{z_{Ln},\;z_{Rn}\right\}<+\frac{W_{P}\left(x\right)}{2},$$ (1)

where we take the *z*-coordinate to define the lateral direction (Fig 1B and 1C), *W*_*P*_(*x*) defines the net width of the walking path, and {*z*_*Ln*_, *z*_*Rn*_} define left and right lateral foot placements for the *n*^th^ step in a sequence of *N* consecutive steps. Walking on a treadmill, as studied here, merely implies *W*_*P*_(*x*) = *W*_*TM*_ ≡ the constant width of the treadmill belt, but Eq (1) applies generally to any walking path with finite width (e.g., Fig 1A, etc.).

Our objective is to identify what strategies people choose to accomplish this very general walking task. A key observation is that *any* sequence of {*z*_*Ln*_, *z*_*Rn*_} that satisfies the inequality of Eq (1) will achieve this. Thus, an infinite number of choices for each *z*_*Ln*_ and *z*_*Rn*_ exists and many strategies could generate feasible sequences of {*z*_*Ln*_, *z*_*Rn*_} that satisfy Eq (1). To identify these strategies, we need to quantify the lateral motion of the walker relative to the path, and the lateral motions of the walker’s feet relative to its body.

While {*z*_*Ln*_, *z*_*Rn*_} define the task goal via Eq (1) and ultimately enact any stepping control strategy, we presume {*z*_*Ln*_, *z*_*Rn*_} are coordinated to achieve some more general goal related to whole body movement. In particular, {*z*_*Ln*_, *z*_*Rn*_} establish where the walker’s body *is* relative to the path [25, 32, 33, 35]. Here, we take this body position on any given step, *z*_*Bn*_, as the midpoint between the two feet (Fig 1B and 1C):

$$z_{Bn}=\frac{z_{Ln}+z_{Rn}}{2}.$$ (2)

This choice of *z*_*Bn*_ approximates the center-of-mass location for steady-state upright walking on level ground [21, 62]. Eq (2) assumes only that stepping movements are symmetrical relative to the center-of-mass, a finding broadly validated in multiple studies of human walking [42, 63–65]. Eq (2) is also consistent with multiple well-established models of pedestrian navigation [52, 54, 55].

In addition to the walker’s location on the path, we must also consider how it *moves* relative to its path. Goal-directed walking and pedestrian navigation require maintaining direction [52, 53] or “heading” [66, 67]. Likewise, the lateral (*z*) component of CoM velocity contributes to subsequent foot placement within each step [18, 33, 46] and in the sagittal plane, humans regulate speed and not position [9, 10]. Here, we thus take Δ*z*_*Bn*_ as a proxy for lateral speed, or equivalently the lateral component of “heading” (Fig 1C):

$$\Delta z_{Bn}=z_{Bn}-z_{B\left(n-1\right)}.$$ (3)

Lastly, regulating step width to maintain lateral balance is also important to walking [16, 21, 23, 68]. Here, we define step width as the difference between the lateral locations of the right and left foot (Fig 1C):

$$w_{n}=z_{Rn}-z_{Ln}.$$ (4)

Thus, from the most basic definition of walking, any biped walker (human, robot, model, etc.) must therefore exhibit some sequence of {*z*_*Ln*_, *z*_*Rn*_, *z*_*Bn*_, Δ*z*_*Bn*_, *w*_*n*_} over consecutive steps. However, by definition these variables are not all independent, as can be readily seen by writing Eqs (2) and (4) in matrix form as:

$$\left[\begin{array}{c} z_{Bn}\\ w_{n} \end{array}\right]=\left[\begin{array}{cc} \frac{1}{2} & \frac{1}{2}\\ -1 & 1 \end{array}\right]\left[\begin{array}{c} z_{Ln}\\ z_{Rn} \end{array}\right]\;\Leftrightarrow \;\left[\begin{array}{c} z_{Ln}\\ z_{Rn} \end{array}\right]=\left[\begin{array}{cc} 1 & -\frac{1}{2}\\ 1 & \frac{1}{2} \end{array}\right]\left[\begin{array}{c} z_{Bn}\\ w_{n} \end{array}\right].$$ (5)

Thus, specifying either [*z*_*Ln*_, *z*_*Rn*_] or [*z*_*Bn*_, *w*_*n*_] fully specifies the other. We therefore take {*z*_*Bn*_, Δ*z*_*Bn*_, *w*_*n*_} to be the biomechanically relevant variables to be regulated and treat {*z*_*Ln*_, *z*_*Rn*_} as effectors that enact this regulation. Like our prior sagittal-plane work [10], strategies that regulate some combination of {*z*_*Bn*_, Δ*z*_*Bn*_, *w*_*n*_} thus define “control templates” to describe step-to-step *regulation* of lateral stepping movements that can then compliment mechanical templates of within-step locomotor biomechanics [57, 58]. We then seek the most parsimonious such strategy that captures the variability and step-to-step dynamical structure we observe in experiments.

### Lateral stepping dynamics in humans

To test different hypothesized control strategies, we re-analyzed data from a prior study in which young healthy human participants walked on a 1.77 m wide motorized treadmill at a pre-determined comfortable walking speed [69, 70]. We measured time series of left (*z*_*L*_) and right (*z*_*R*_) lateral foot placements. We used Eqs (2)–(4) to calculate corresponding time series of body positions (*z*_*B*_), heading (Δ*z*_*B*_), and step widths (*w*), which we then analyzed.

To quantify variability, we computed standard deviations for each time series. To quantify temporal correlations across consecutive steps, we computed scaling exponents, *α*, using Detrended Fluctuation Analysis (DFA) [9, 71] (see Methods). An *α* > ½ indicates statistical *persistence* (deviations in either direction are more likely to be followed by deviations in the same direction). An *α* < ½ implies *anti*-persistence (subsequent deviations are more likely to be in the opposite direction). An *α* = ½ indicates uncorrelated fluctuations (subsequent deviations are equally likely to be in either direction). A value of *α* = 1½ indicates brown noise (i.e., integrated white noise), equivalent to Brownian motion. In the context of control, variables *not* tightly regulated typically exhibit strong persistence (*α* >> ½). Conversely, variables that *are* more tightly regulated typically exhibit approximately uncorrelated fluctuations (*α* ≈ ½) [9, 10, 12].

**Fig 2:**
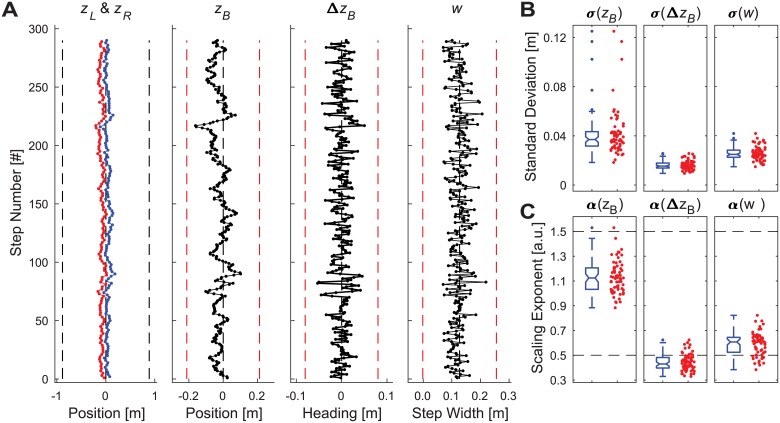
Experimental values of primary stepping variables. A) Example time series data for a representative trial from a typical participant. Each plot shows 290 consecutive steps of left (*z*_*L*_; red) and right (*z*_*R*_; blue) foot placements, body position (*z*_*B*_), heading (Δ*z*_*B*_), and step width (*w*). For the stepping plot (*z*_*L*_; *z*_*R*_), black vertical dashed lines indicate the lateral edges of the treadmill (±0.885 m). For the other time series plots (*z*_*B*_, Δ*z*_*B*_, and *w*), red vertical dashed lines indicate ±5 standard deviations, as determined from the average of the standard deviations of all participants. B) Standard deviation (*σ*) values for all trials for all participants for each variable (*z*_*B*_, Δ*z*_*B*_, and *w*). C) DFA scaling exponent (*α*) values for all trials for all participants for each variable (*z*_*B*_, Δ*z*_*B*_, and *w*). For (B) and (C), each subplot shows a summary boxplot (blue–left) indicating the median, 1^st^ and 3^rd^ quartiles, and whiskers extending to 1.5× the inter-quartile range, with values beyond that range shown as individual data points. Each subplot also shows individual data points (red dots–right) indicating all individual trials for all participants. These experimental data were aggregated across 65 total trials of 290 steps each, as obtained from 13 participants (5 trials each).

All of the candidate stepping variables, {*z*_*Bn*_, Δ*z*_*Bn*_, *w*_*n*_}, and also foot placements, {*z*_*Ln*_, *z*_*Rn*_}, fluctuated (e.g., Fig 2A), with magnitudes of variability well above the level of measurement noise (Fig 2B). Fluctuations in lateral body position (*z*_*B*_) exhibited strong statistical persistence (*α* >> ½). Fluctuations in heading (Δ*z*_*B*_) exhibited mostly statistical anti-persistence (*α* < ½), consistent with heading being the first-difference of position (Fig 2C). Fluctuations in step width (*w*) exhibited weak statistical persistence (½ < *α* << 1½).

Given that all variables exhibited both substantial variability and varying degrees of statistical persistence, one cannot conclude from these data alone which variable (or combination of variables) were or were not regulated. Indeed, we might be tempted to conclude that Δ*z*_*B*_ and *w* were more tightly regulated than *z*_*B*_ because they both exhibited *α* ≈ ½ [9, 10, 12]. However, any such inference would be incorrect. As we demonstrate, such a conclusion would succumb to the formal logical fallacy of “affirming the consequent” [72]: i.e., while processes that are tightly regulated typically yield α ≈ ½, not all processes that exhibit *α* ≈ ½ are tightly regulated. What is needed are appropriate computational models that can accurately predict these variability and statistical persistence relationships and indicate what gives rise to them.

### Uni-objective stepping regulation

We consider first the simplest such stepping control strategies, namely those that regulate only one of the variables (Eqs (2)–(4)) to maintain some constant value of that variable. The strategy “stay in the middle of the path” implies one would adjust consecutive foot placements to maintain *z*_*Bn*_ = *z*_B_* ≡ Constant. We note that other reasonable choices for *z*_*Bn*_ different from Eq (2) exist. Each would yield a different corresponding *z*_B_*, but would not change the step-to-step dynamics quantified here. Likewise, because we can define the origin of our coordinate system (Fig 1) at any point we choose, we could always take *z*_B_* = 0, but we retain the more general form for now. The strategy “maintain heading” (regardless of lateral position) implies that one would try to maintain Δ*z*_*Bn*_ = Δ*z*_B_* ≡ Constant. Here, Δ*z*_B_* = 0 would represent the goal to walk “straight ahead” (i.e., with no lateral deviation), but again we retain the more general form for now. Lastly, the strategy “maintain step width” (e.g., as in [16, 22, 23]) implies that one would try to maintain *w*_*n*_ = w* ≡ Constant (regardless of absolute position or heading). Here, we would take w* = 〈*w*〉 ≡ some average (or typical) step width (e.g., for an individual or group, etc.) as the desired step width to maintain.

Each of these strategies can be defined in terms of a scalar “*goal function*” [6, 7, 12] in each variable separately, each having the same general form:

$$F_{q}=q_{n}-q^{*},$$ (6)

where *q* ∈ {*z*_*B*_, Δ*z*_*B*_, *w*} and the goal is to drive *F*_*q*_ → 0 (i.e., minimize errors with respect to the goal). For each candidate control variable, we write a corresponding state-update equation governing the regulated step-to-step dynamics as a 1D map in *q*:

$$q_{n+1}=q_{n}+g\left(1+\sigma_{m}\nu_{m}\right)u_{q}\left(q_{n}\right)+\sigma_{a}\nu_{a},$$ (7)

where *q* is the controller “state” being regulated, *u*_*q*_(*q*_*n*_) is the relevant control input, *σ*_*m*_*ν*_*m*_ and *σ*_*a*_*ν*_*a*_ are multiplicative and additive noise terms, respectively, and *g* is an additional gain (introduced in our previous work: [9, 10]) that could be used to tune the regulator away from the derived optimal control. Here, no such tuning was necessary, so we set *g* = 1 for all subsequent analyses. Single variable regulators such as in Eq (7) thus allow the remaining unregulated variables to “drift” under the action of motor noise.

Following prior work [9, 10, 12], these controllers were derived analytically (see Methods) as stochastic optimal single-step controllers with direct error feedback, following the Minimum Intervention Principle [3, 4]. This yielded the following stochastically optimal control input, *u*_*q*_, as a function of the current state, *q*_*n*_:

$$u_{q}\left(q_{n}\right)=-\left[\frac{1}{\left(1+\sigma_{m}^{2}+\left(\gamma /\alpha \right)\right)}\right]\left(q_{n}-q^{*}\right),$$ (8)

We used these models to simulate walking trials that we then compared directly to experimental data from humans. Each such model depends on three parameters (*σ*_*a*_, *σ*_*m*_, and *γ*/*α*). We conducted parameter sensitivity analyses (see S1 Appendix) across wide ranges of each of these parameters to determine if any of these candidate uni-objective control models could adequately replicate the experimental findings of Fig 2.

**Fig 3:**
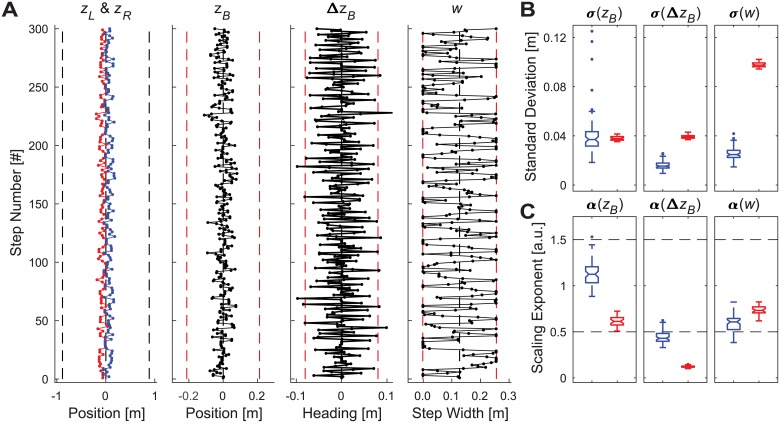
Typical model simulation results for biomechanically constrained position control. A) Example time series data for a single representative trial. The time series plotted, axes, and axis limits are all the same as in Fig 2. B) Standard deviation (*σ*) values and (C) DFA scaling exponent (*α*) values for all trials for each variable (*z*_*B*_, Δ*z*_*B*_, and *w*). Each subplot in (B) and (C) shows boxplots (blue–left) and for 30 representative simulated trials from the model (red–right). All boxplots were constructed in the same manner as described in Fig 2. This model failed to replicate experimental findings from humans, as did all other uni-objective control models across all parameter ranges tested (see S1 Appendix for complete details).

None of the uni-objective control models came close to capturing the behavior observed experimentally, regardless of parameter choices (see S1 Appendix for details). Nearly all simulations either regularly stepped off the prescribed path, or took biomechanically unrealistic steps, or both. We therefore also imposed additional biomechanically realistic constraints to each control model (see S1 Appendix for details) to ensure they did not either walk off the path (here, the treadmill: *z*_*Max*_ = ±0.885 m), or take steps that were unrealistically too wide or too narrow (defined here as no more than ±5 standard deviations, based on experimental means; Fig 2). All of these control models fully satisfied (by construction) the requirements of the walking task (Eq 1) and did so by taking steps that were biomechanically feasible [34, 62, 73]. Nevertheless, all of the model configurations tested (e.g., Fig 3) exhibited step-to-step dynamics that were both qualitatively and quantitatively substantially different from humans (see S1 Appendix for details) and thus failed to replicate the lateral stepping behavior observed in experiment (Fig 2).

### Multi-objective stepping regulation

Because no uni-objective controller model (with or without added biomechanical constraints) adequately replicated experimental findings, we next considered *multi*-objective control of 2 candidate parameters. The general 2-state vector-valued goal function [6, 7, 12] for any of these becomes:

$$F=\left[\begin{array}{c} q_{1n}-q_{1}^{*}\\ q_{2n}-q_{2}^{*} \end{array}\right],$$ (9)

where *q*_1_ and *q*_2_ are any two distinct variables selected from *q*_1,2_ ∈ {*z*_*B*_, Δ*z*_*B*_, *w*} and the goal is again to drive **F** → 0. The corresponding state-update equation becomes:

$$\left[\begin{array}{c} q_{1\left(n+1\right)}\\ q_{2\left(n+1\right)} \end{array}\right]=\left[\begin{array}{c} q_{1n}\\ q_{2n} \end{array}\right]+\left[\begin{array}{cc} g_{1} & 0\\ 0 & g_{2} \end{array}\right]\left[\begin{array}{cc} 1+\sigma_{1m}\nu_{1m} & 0\\ 0 & 1+\sigma_{2m}\nu_{2m} \end{array}\right]\left[\begin{array}{c} u_{q1}\\ u_{q2} \end{array}\right]+\left[\begin{array}{c} \sigma_{1a}\nu_{1a}\\ \sigma_{2a}\nu_{2a} \end{array}\right],$$ (10)

where the *q*_1,2_ are the controller states being regulated. We then followed the same analytical derivation process [9, 10, 12] (see Methods), which yielded the following stochastically optimal control input:

$$\left[\begin{array}{c} u_{q1}\left(q_{1n}\right)\\ u_{q2}\left(q_{2n}\right) \end{array}\right]=\left[\begin{array}{cc} -1/\left(1+\sigma_{1m}^{2}+\left(\gamma_{1}/\alpha_{1}\right)\right) & 0\\ 0 & -1/\left(1+\sigma_{2m}^{2}+\left(\gamma_{2}/\alpha_{2}\right)\right) \end{array}\right]\left[\begin{array}{c} q_{1n}-q_{1}^{*}\\ q_{2n}-q_{2}^{*} \end{array}\right].$$ (11)

Both the state update equation (Eq (10)) and control inputs (Eq (11)) were chosen to have no coupling between their *q*_1_ and *q*_2_ components (see Methods). Thus, at each new step, each individual optimal controller (for *q*_1_ and *q*_2_ separately) predicts a different foot placement for the next foot. As one cannot place one’s foot in two places at once, the net result (i.e., final predicted foot placement) becomes a weighted average of the foot placements predicted by each of the uni-variate models for *q*_1_ and *q*_2_ separately.

We used these multi-objective models to simulate walking trials that we then compared directly to experimental data from humans (Fig 2). We conducted parameter sensitivity analyses (see S2 Appendix) across wide ranges of each of the governing parameters to determine if any of these candidate multi-objective control models could adequately replicate the experimental findings of Fig 2. Here, we emphasized varying the simulation parameter (*ρ*) that specified the weighted average between *q*_1_ control and *q*_2_ control (see S2 Appendix for details).

Simulations that regulated combinations of position and heading (i.e., [*q*_1_, *q*_2_] = [*z*_*B*_, Δ*z*_*B*_]) failed to replicate human stepping dynamics, mainly by regularly violating reasonable step width limits (Fig. S2-2 in S2 Appendix). Across a substantial range of the parameter space tested, simulations that regulated combinations of heading and step width (i.e., [*q*_1_, *q*_2_] = [Δ*z*_*B*_, *w*]) did successfully achieve the task goal (Eq (1)) and did so using biomechanically reasonable step widths (Fig. S2-4 in S2 Appendix). Thus, many of these simulations predicted stepping strategies humans *could* have used to accomplish the task. However, *all* of these simulations yielded variability and statistical persistence of lateral position (*σ*(*z*_*B*_) and *α*(*z*_*B*_)) that far exceeded experimental values (Fig. S2-4 in S2 Appendix). Thus, all simulations regulating heading and step width failed to replicate human stepping dynamics, even though this strategy was physiologically feasible.

**Fig 4:**
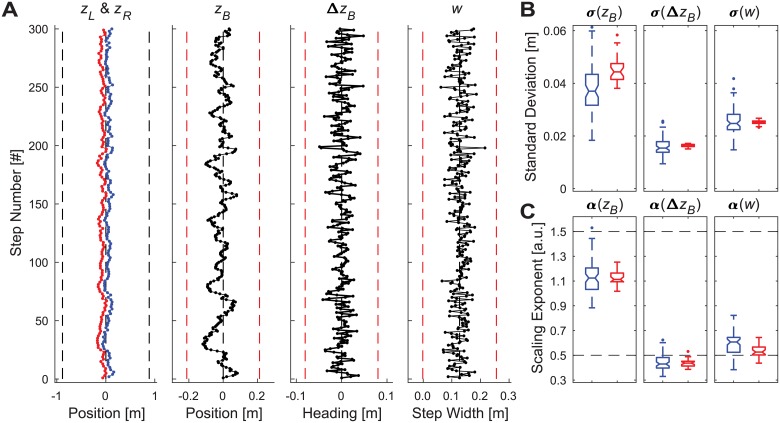
Typical model simulation results for multi-objective position-step width control. A) Example time series data for a single representative trial simulated for baseline parameter values (see S2 Appendix) and a control proportion that was weighted at 93% step width / 7% position control. The time series plotted, axes, and axis limits are all the same as in Fig 2. B) Standard deviation (*σ*) and (C) DFA scaling exponent (*α*) values for all trials for each variable (*z*_*B*_, Δ*z*_*B*_, and *w*). Each subplot in (B) and (C) shows boxplots for the experimental data (blue–left; from Fig 2) and for 30 representative simulated trials from the model (red–right) using the same parameter values as in (A). All boxplots were constructed in the same manner as described in Fig 2.

Simulations that regulated combinations of position and step width (i.e., [*q*_1_, *q*_2_] = [*z*_*B*_, *w*]) also failed to replicate human stepping dynamics over a wide range of *ρ* (0% ≤ *ρ* < ~89% and *ρ* > ~97%; see Supplement S2; Fig. S2-6). However, for baseline parameter values, when the distribution of control was weighted to favor ~93% step width control, vs. ~7% position control, these simulations did replicate the basic statistical properties of human stepping dynamics (namely, both the standard deviations (*σ*) and statistical persistence (*α*) for all of the specified variables (*z*_*B*_, Δ*z*_*B*_, and *z*_*B*_) (Fig 4; same as Fig. S2-5 in S2 Appendix).

**Fig 5:**
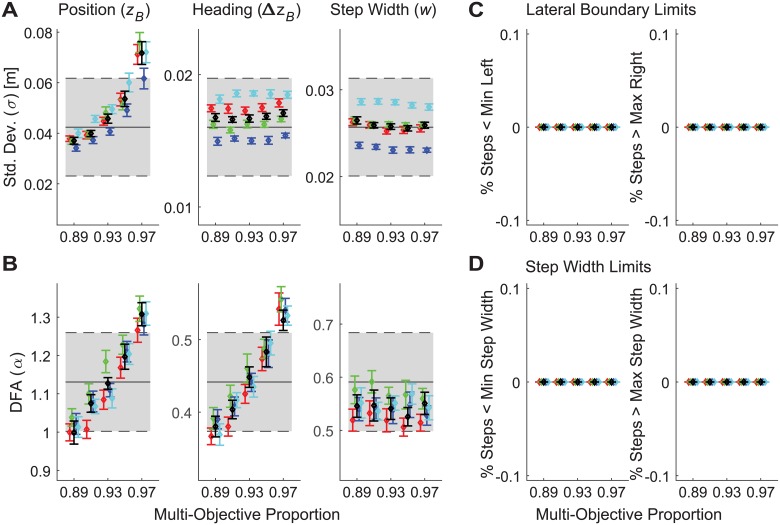
Parameter sensitivity results for multi-objective position-step width control. A) Standard deviations for each primary output variable (*z*_*B*_, Δ*z*_*B*_, and *z*_*B*_). B) DFA scaling exponents (*α*) for each primary output variable. In both (A) and (B), horizontal gray bands indicate the mean ± 1SD band exhibited by humans for that variable to indicate the range of values observed experimentally. For display purposes, all vertical axes are scaled to the mean ± 2SD band exhibited by humans. C) Percentage of steps (*z*_*L*_ and *z*_*R*_) taken in each simulated trial that exceeded the Lateral Boundary Limits (±0.885 m): i.e., stepped off the treadmill. D) Percentage of steps that exceeded Step Width Limits (±5σ as determined from experimental data; equivalent step width range: −0.15 cm to +25.54 cm), reflecting biomechanically unrealistically wide or narrow steps (see S1 Appendix for details). Stepping data shown here were simulated at multi-objective proportions from 89% step width (11% position) control, every 2% up to 97% step width (3% position) control. Black symbols indicate ‘baseline’ parameter values (see S2 Appendix): *σ'*_*a*_ based on experimental values, *σ'*_*m*_ = 0.1∙*σ'*_*a*_, and (*γ/α*)*'* = 0.1. Red and Green symbols indicate these same baseline parameter values, except *γ/α* = 0.0 and 0.2, respectively. Blue and Cyan symbols indicate these same baseline parameter values, except *σ*_*a*_ = 0.9∙*σ'*_*a*_ and 1.1∙*σ'*_*a*_, respectively.

Indeed, more refined parameter sensitivity analyses across a narrower range of 89% ≤ *ρ* ≤ 97% (Fig 5) yielded a range of solutions weighted strongly in favor of step width control that successfully replicated human stepping dynamics (Fig 2) and were also robust to variations in additive noise (*σ*_*a*_) and cost function weights (*γ*/*α*) (Fig 5A). For all parameter variations tested across the range 89% ≤ *ρ* ≤ 97%, no simulation ever took any steps that exceeded either the lateral boundary limits (i.e., stepped off the treadmill; Fig 5C) or the step width limits (i.e., took excessively wide or narrow steps; Fig 5D). Thus, across all of these cases, this model successfully achieved the walking task (Eq (1)), did so by taking biomechanically feasible steps, and also successfully replicated all of the same stepping dynamics observed experimentally in humans.

Measures of variability (e.g., Fig 5A, etc.) quantify average magnitudes of differences across all steps, but do not indicate how stepping is executed dynamically over *time*. DFA analyses (e.g., Fig 5B, etc.) quantify statistical persistence (average step-to-step linear dependence) across time [9, 71], but independent of the *magnitude* of the variance. Thus, neither of these calculations (σ or α) quantifies how the proportionality of subsequent corrections (in time) of step-to-step fluctuations might differ with the magnitude of those initial fluctuations. Conversely, the Minimum Intervention Principle [3] and *N*-step capturability concept [44] both suggest that corrections to errors could well vary nonlinearly with the magnitudes of those errors: i.e., one could readily ‘ignore’ small errors and correct only larger ones. Neither σ nor α would capture any such dependence. Therefore, we conducted a third *independent* set of analyses to verify these findings [10].

**Fig 6:**
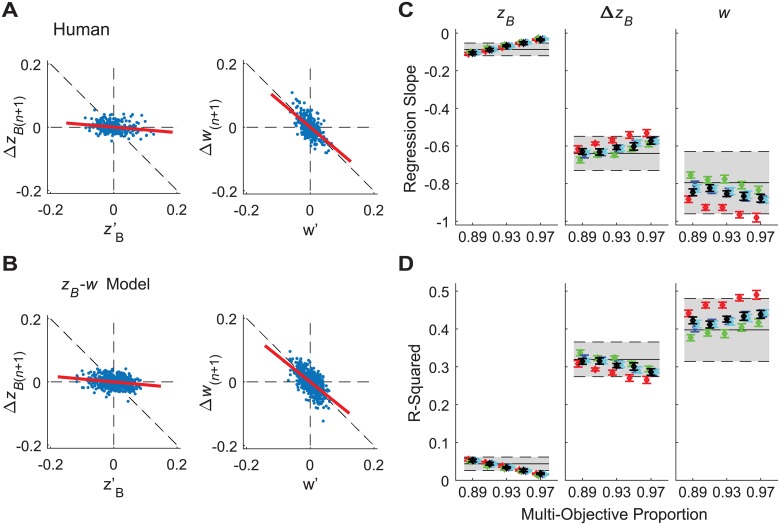
Direct control analysis results for multi-objective position-step width control. A-B) Example plots of how errors in relative position (*z'*_*Bn*_) and relative step width (*w*') were corrected on subsequent strides (Δ*z*_*B*(*n*+1)_ and Δ*w*_(*n*+1)_). Data are shown for (A) one typical experimental trial from typical human participant and for (B) one typical trial from the multi-objective position-step width controller adopting baseline parameter values (see Fig 6). C) Linear regression slopes for each corresponding relationship for each primary output variable (*z*_*B*_, Δ*z*_*B*_, and *z*_*B*_). D) Corresponding linear correlation (r^2^) values for each linear regression for each primary output variable. In both (C) and (D), horizontal gray bands indicate the mean ± 1SD band exhibited by humans for that variable to indicate the range of values observed experimentally. In both (C) and (D), horizontal axis limits and symbol / color designations are the same as shown in Fig 5.

If steps are adjusted at each step based on the previous step (as they are designed to be in our models; Eqs (7) and (10)), we would hypothesize that a stepping regulator trying to maintain some constant average value of any $q^{*}\equiv \bar{q}$ (Eqs (6) & (9)), when observing any deviation on a given step (i.e., ${q^{\prime }}_{n}=q_{n}-\bar{q}$), should correct that deviation on the subsequent step by making a corresponding change, Δ*q*_n+1_ = *q*_n+1_ − *q*_n_, in the opposite direction [10]. For each simulated and experimental (human) data set, we constructed plots of Δ*q*_n+1_ vs. *q'*_n_ for each *q* ∈ {*z*_*B*_, Δ*z*_*B*_, *w*}. We computed the linear slopes (using least-squares regression) and strength of correlation (r^2^) for each corresponding relationship [10]. We assumed our simulations, which enacted precisely this form of control, would exhibit evidence of strong linear control of step width (*w*), reflected by slopes close to −1 with high correlation, but weak linear control of position (*z*_*B*_), reflected by slopes close to 0 with very low correlation. We hypothesized that if humans exerted step-to-step control in a similar manner, they would exhibit results consistent with our simulations. Indeed, this was precisely the case (Fig 6). All of the same simulations that precisely matched human stepping dynamics in Fig 5 (above) also just as precisely matched the strength of direct step-to-step corrections of all three time series variables (Fig 6).

Together, the results of Figs 5 and 6 make clear that the multi-objective position–step width control model accurately replicates the basic step-to-step dynamics of the fundamental variables {*z*_*Bn*_, Δ*z*_*Bn*_, *w*_*n*_}. Of course, these models not only need to regulate these variables appropriately, but should also generate appropriate stepping movements of the feet themselves {*z*_*Ln*_, *z*_*Rn*_}, as these are the effectors that enact this regulation. Therefore, as a final confirmation of the preceding results, we assessed how the above dynamics then mapped onto the stepping dynamics of {*z*_*Ln*_, *z*_*Rn*_}, which is easily accomplished using Eq (5).

**Fig 7:**
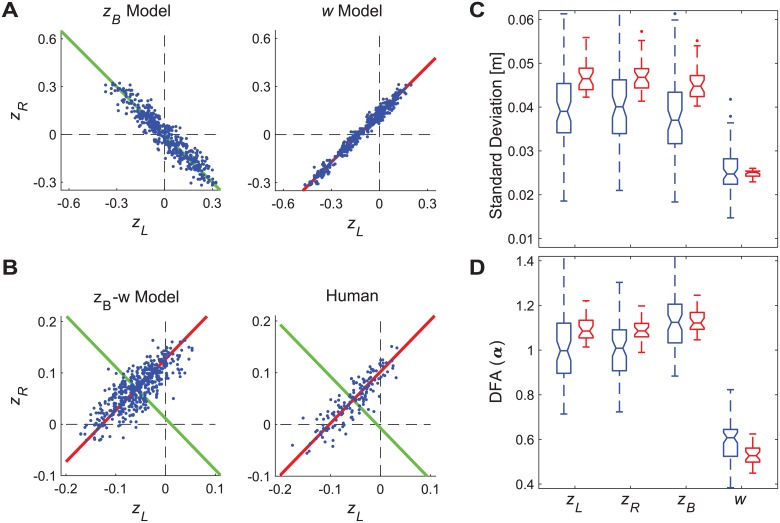
Projection of [*z*_*B*_, *w*] control variables onto the [*z*_*L*_, *z*_*R*_] stepping plane. A) Example plots of stepping data for simulations of uni-objective controllers projected onto the [*z*_*L*_, *z*_*R*_] plane (see Eq (5)). One typical simulation trial each is shown for controllers regulating either position (*z*_*B*_) only (left) or step width (*w*) only (right), each simulated using baseline parameter values (see S1 Appendix). In each plot, diagonal lines indicate the *z*_B_* = constant (green, left) or w* = constant (red, right) GEM’s, as determined from the average position or step width (respectively) exhibited on that trial. All combinations of [*z*_*L*_, *z*_*R*_] that lie along either GEM equally satisfy the respective goal. B) Example plots of stepping data for one typical simulated trial from the multi-objective position–step width (*z*_*B*_-*w*) controller, simulated using baseline parameter values (see S2 Appendix) and for one typical experimental trial. For each plot, the *z*_B_* and w* GEM’s were determined from the average position and step width exhibited on that trial. C) Standard deviation (*σ*) and (D) DFA scaling exponent (*α*) values for all trials for left (*z*_*L*_) and right (*z*_*R*_) steps, body position (*z*_*B*_), and step width (*w*) for humans (blue) and for the multi-objective position–step width controller, simulated using baseline parameter values (red). All boxplots were constructed in the same manner as described in Fig 2. Note that as all variables are in units of meters, variability measures can be compared directly, as coordinate dependence is not an issue here. All simulation values are well within the range of experimental results. Thus, the multi-objective position–step width control model captured the observed left/right stepping dynamics in [*z*_*L*_, *z*_*R*_] as it regulated [*z*_*B*_, *w*].

The goals to maintain constant *z*_*Bn*_ = *z*_B_* and *w*_*n*_ = w*, when considered individually, each yield linear GEMs that are orthogonal to each other in the [*z*_*L*_, *z*_*R*_] plane (Fig 7A). For the multi-objective position–step width control models, the [*z*_*Bn*_, *w*_*n*_] dynamics (i.e., including both variability and temporal correlation structure) maps into [*z*_*Ln*_, *z*_*Rn*_] dynamics precisely as defined by Eq (5). We thus predicted that if humans also adopted control consistent with this policy, their experimental [*z*_*Bn*_, *w*_*n*_] dynamics would also map into [*z*_*Ln*_, *z*_*Rn*_] dynamics in the same way as in the models. Indeed, this was precisely the case (Fig 7B and 7C), further supporting the validity of these multi-objective position–step width control models.

## Discussion

Human walking exhibits considerable variability [14, 74] due to both physiological noise [2] and inherent redundancy [3, 6]. Experimental data alone (e.g., Fig 2) yield no specific insights into which stepping variables (Fig 1C) humans may and/or may not regulate during walking. Thus, appropriate computational models are critical to disambiguate the various possibilities. Existing models of how people regulate lateral foot placement achieve either continuous dynamic balance control [16, 46], or the equifinality of foot placement [18, 44] that people also exhibit [9, 10, 15], but not both. Moreover, existing models that consider only within-step foot placement [16, 32–35, 46] do not capture how humans regulate movements from *step to step* to navigate the environments [52–54] they walk in [27, 28, 51]. The present work extends a theoretical and computational framework [6–8, 12] that previously identified how humans regulate stride-to-stride movements in the sagittal plane [9, 10, 15, 47]. Here, we present new goal functions that posed hypotheses about how humans might regulate *lateral* stepping movements. We developed and implemented predictive models of lateral stepping dynamics that we then used to test those hypotheses against experimental data.

Simple mechanical models have successfully elucidated many fundamental aspects of locomotion dynamics (e.g., [16, 75–77]). These models represent biomechanical “templates” [57–59] for the most basic mechanics and dynamics of any biped (human, robot, model, etc.) and reveal how those biomechanics shape locomotor behavior. Similarly, the stepping models presented here and in our previous work [9, 10] act as “control templates” that serve as minimal models to describe how movements of those mechanical templates (e.g., [16, 75–77]) should be *regulated* from step to step if they are to match human behavior. Our models show how fundamental stepping variables can be regulated to satisfy some particular goal-directed strategy (e.g., “stay in the middle of the path”, “maintain heading”, “maintain step width”, etc.). In this sense, these control templates are symbiotic with mechanical templates. To determine how people walk in their environment [52–54] requires complimentary descriptions both of what governs walking movements biomechanically within each step and also of how the nervous system regulates those movements from step-to-step to achieve specific goal-directed walking tasks. In a parallel way that mechanical templates can be “anchored” [57–59] within more elaborate, higher-dimensional mechanical models, so too could these simple “control templates” be anchored hierarchically within more elaborate neurophysiological control models (e.g., [78, 79], etc.).

Our uni-objective control models (Fig 3 and S1 Appendix) yielded clear evidence of the inherent redundancies involved in regulating any one variable. Several configurations of unconstrained uni-objective control could achieve the required walking task (Eq (1)) with biomechanically feasible stepping movements. All of them could be made to do so by adding appropriate constraints (e.g., Fig 3). However, *none* of the uni-objective control models tested (S1 Appendix) yielded stepping dynamics comparable to humans. Likewise, multiple configurations of various multi-objective control models (Supplement 2) could achieve the required task goals with feasible stepping movements, but most of these likewise failed to replicate human stepping dynamics. However, over a somewhat narrow range, multi-objective control models that strongly prioritized regulating step width while allowing some degree of lateral position control did successfully replicate all of the relevant step-to-step dynamics observed in humans (Figs 4 and 5). Independent analyses confirmed that this control was consistent with linear error correction (Fig 6) and also replicated step-to-step dynamics of foot placement (Fig 7). Thus, in contrast to how humans regulate sagittal plane stepping movements [9, 10, 40, 47], the regulation of lateral stepping movements is inherently *multi*-objective and balances task-specific trade-offs between competing task goals.

In the context of control, variables not tightly regulated [9, 12] typically exhibit stronger statistical persistence (*α* >> ½), tightly regulated variables typically exhibit fluctuations with *α* ≈ ½, and variables that are ‘over-controlled’ exhibit fluctuations with *α* < ½ [9, 12]. However, here we show this is not always the case. Here for example, Δ*z*_*B*_ is the first-difference of *z*_*B*_ (Eq (3)) and it is this relationship that determines how *α*(Δ*z*_*B*_) is related to *α*(*z*_*B*_). In the uni-objective models, controlling *z*_*B*_ (Figs. S1-1 & S1-2 in S1 Appendix) thus yields *α*(*z*_*B*_) ≈ ½ as expected, but *α*(Δ*z*_*B*_) << ½ not because Δ*z*_*B*_ is being ‘over-controlled’, but because Δ*z*_*B*_ is the first-difference of *z*_*B*_. Controlling Δ*z*_*B*_ (Figs. S1-3 & S1-4 in S1 Appendix) yields *α*(Δ*z*_*B*_) ≈ ½ as expected, but *α*(*z*_*B*_) ≈ 1½ because integrating the uncorrelated Δ*z*_*B*_ yields Brownian motion of *z*_*B*_. Lastly, controlling *w* (Figs. S1-5 & S1-6 in S1 Appendix) yields both *α*(*z*_*B*_) ≈ 1½ and *α*(Δ*z*_*B*_) ≈ ½ when neither variable is being controlled. Thus, inferring that Δ*z*_*B*_ is ‘tightly regulated’ because we observed *α*(Δ*z*_*B*_) ≈ ½ experimentally (Fig 2), without considering how Δ*z*_*B*_ relates to *z*_*B*_ would constitute “affirming the consequent” [72]: i.e., just because *A* → *B*, does not mean *B* → *A*. Thus, both the experimental findings (Fig 2) and model results (S1 and S2 Appendices) are consistent with how Δ*z*_*B*_ and *z*_*B*_ are related. However, only the modeling results demonstrate how the step-to-step fluctuation dynamics (as quantified by *α*) reflect which variables are (and are not) being regulated.

Several recent experimental studies have demonstrated how center of mass state (lateral displacement and velocity) at mid stance predicts subsequent lateral foot placement *within a given step* [32–37]. However, such correlations may not reflect within-step “control”, but instead result mainly from passive dynamics [25, 33]. They also do not consider other possible within-step mechanisms of balance control such as modulation of ankle torques, push-off, etc. [80, 81]. The present work both extends and compliments that work in at least two critical ways. First, we directly identify how humans regulate stepping movements *across consecutive steps*. Within-step models and/or analyses cannot capture the between-step regulation of stepping movements that is required to achieve *goal-directed* walking tasks [9, 10, 26–28, 38–40]. Wang et al. [33] found that position on the treadmill (which they called ‘station keeping’) contributed to lateral foot placement, but dismissed this because it accounted for only a very small amount of the total variance. Here, we show definitively that even though lateral position is regulated much more weakly than step width (by roughly the same percent as in [33]), this contribution remains critical because without it, the walker would eventually drift off of the path (to one side or the other). Such effects could not be appreciated from only within-step analyses, such as those in [32–37]. Second, we do this in a way that directly identifies how step-to-step regulation achieves *goal-directed* walking [52–54], which is a necessary component of any comprehensive understanding of how humans (or other bipeds) walk within any environmental context [27, 28, 51].

A critical question in computational neuroscience is to determine how the nervous system makes accurate goal-directed movements in the face of physiological noise [1, 2] and redundancy [3–5]. The Minimum Intervention Principle [3, 4] suggests the nervous system should correct only deviations that adversely affect task performance and ignore variations that do not. We previously identified evidence for such control in regulating sagittal plane stepping [9, 10, 15, 40, 47] that was independently verified by other studies [48–50]. The present work extends these ideas to regulation of lateral stepping. Here, we likewise identified strong evidence of ‘MIP-type’ control in several respects: the clear redundancy between variables (Fig 3 and S1 Appendix), replication of experimental findings by regulating only 2 candidate variables (Figs 4–7 and S2 Appendix), and prioritization of step width control over lateral position control (Figs 5 and 6). However, our findings also reveal limits to the MIP concept. Given the wide (1.77 m) path (treadmill belt) participants walked on, they easily *could* have ignored small deviations in lateral position, instead making corrections only for very large deviations (i.e., near the lateral edges of the treadmill). Likewise, given the capture *region* predictions of [44] and/or *margin* of stability predictions of [18], participants *could* have similarly ignored small deviations in step width, instead correcting only much larger deviations. Implementing any such strategy would have led to nonlinear (e.g., piece-wise linear, “deadband”, cubic, etc.) distributions in the plots of Fig 6A and 6B, reflecting greater proportional correction of larger errors compared to smaller ones. However, the linear relationships shown in Fig 6 clearly demonstrate that participants did not do this. Instead, participants strongly corrected both small and large deviations in step width similarly and weakly corrected both small and large deviations in lateral position similarly.

Still other viable alternative control strategies exist to regulate lateral stepping movements while walking. For example, given the redundancies clearly available in both step width and lateral position, there is no physiological necessity to correct errors over only one step, as our models do. A walker (human, robot, model, etc.) could instead correct errors more gradually over multiple steps, as described for example in [44], and still achieve the task goal (Eq (1)) equally as well as the models presented here. However, implementing control one step at a time is mechanically sufficient to maintain stable walking [16, 41, 42, 46] and our work is consistent with those ideas. Our models for regulating lateral stepping are also consistent with our prior models that regulated sagittal plane stepping [9, 10] and complimentary to experimental findings of within-step control of foot placement [32, 33, 36, 80, 81]. Even when navigating far more complex environments containing many targets/obstacles, visual information about these impediments is acquired only 1–3 steps in advance [27, 28]. Regulating movements only one (or a very few) step(s) at a time allows humans to efficiently navigate complex environments using a noisy neuromotor system [1, 2], where trying to predict motor outcomes farther into the future would not be practical.

The stepping regulation simulations presented (Figs 4–7) hold for the context studied here: i.e., walking on a level surface along a straight path of constant width (i.e.: *W*_*P*_(*x*) = *W*_*TM*_ ≡ constant). These simulations demonstrate what step-to-step regulation is minimally required achieve this task: i.e., regulate ‘mostly step width but some lateral position’ (Figs 5, 6 and 7). Under different task conditions, however, we would fully expect other combinations of regulation to arise. For example, we might expect people to regulate lateral position more tightly when walking on a narrow path [73] or on fixed lines on a treadmill (e.g., as in [34]), etc. People might regulate both position and step width more tightly when subjected to lateral perturbations [23]. People might regulate heading [52, 53, 66] when walking on curved paths, etc. Thus, it is critical to understand that the final control policies revealed here (Figs 5 and 6) do not represent “the” controllers for regulating lateral stepping. Quite the opposite: walking successfully in a complex world [28, 51] requires flexibility in how we regulate our stepping movements. Indeed, different task conditions (those mentioned above and others) would introduce new and/or different task *goals*. Thus, the most important contribution of this work is to present a coherent goal-directed *framework* [7, 12] that can generate empirically testable hypotheses about how humans might regulate stepping movements across a wide *range* of walking tasks and/or conditions.

## Methods

### Ethics statement

Prior to participating, all participants signed informed consent statements approved by the Institutional Review Boards of both Brooke Army Medical Center and The University of Texas at Austin.

### Experimental participants

We compared model predictions to human experimental data taken from the same data set as analyzed in [69, 70]. Thirteen young healthy adults participated (Table 1). All participants were screened to exclude anyone who reported any history of orthopedic problems, recent lower extremity injuries, any visible gait anomalies, or were taking medications that may have influenced their gait.

**Table 1:** Participant characteristics. All values except Sex are given as Mean ± Standard Deviation. Walking speeds shown are the participants’ actual speeds [m/s]. These corresponded to the same pre-designated non-dimensional walking speed (Froude speed ≡ *Fn* = 0.16) [69, 70] for all participants.

Characteristic:	Value:
Sex	10 M / 3 F
Age [yrs]	24.8 ± 6.92
Body Height [m]	1.75 ± 0.08
Body Mass [kg]	79.3 ± 11.56
Body Mass Index (BMI) [kg/m^2^]	26.0 ± 3.96
Leg Length [m]	0.95 ± 0.05
Walking Speed [m/s]	1.22 ± 0.03

### Experimental protocol and data processing

Experimental protocols were described in detail previously [69, 70]. In brief, all participants walked on a 1.77m wide treadmill in a Computer Assisted Rehabilitation Environment (CAREN) virtual reality system (Motek, Amsterdam, Netherlands). Each participant walked at a constant speed (Table 1), non-dimensionally scaled to their leg length [69]. Following a 6-minute acclimation period, each participant completed five 3-minute walking trials with normal optic flow and visual scene movement matched to the treadmill belt speed.

Kinematic data were collected at 60 Hz using a 24-camera motion capture system (Vicon Motion Systems, Oxford, UK) and 57 reflective markers (Wilken et al., 2012). These kinematic data were post-processed using Vicon Nexus and Visual3D (C-Motion Inc., Germantown, MD). For the analyses presented here, only foot and pelvis marker data were used.

Heel strikes were determined using a velocity-based detection algorithm [82]. To increase precision of these calculations, raw marker data were first interpolated from 60 Hz to 600 Hz using a piecewise cubic spline interpolation [40, 47]. For each step, lateral foot placements (*z*_*L*_ and *z*_*R*_) were taken as the lateral location of the heel marker at that step (Fig 1C). From these foot placements, body position (*z*_*B*_), heading (Δ*z*_*B*_), and step width (*w*) were computed using Eqs (2)–(4) (Fig 1C). For consistency, only the first 290 consecutive steps from each trial (e.g., Fig 2A) were analyzed.

### Uni-objective stochastic optimal regulator models

We consider three potential stepping control strategies (Fig 1C): while walking, try to maintain approximately constant absolute position (*z*_*B*_) of the body on the path, constant lateral speed or heading (Δ*z*_*B*_), or constant step width (*w*). Each of these strategies yields a *goal function* [6, 7, 12] in each variable separately, each having the same form (see also Eq (6)):

$$F_{q}=q_{n}-q^{*}.$$ (12)

where *q* ∈ {*z*_*B*_, Δ*z*_*B*_, *w*}. The relevant step-to-step dynamics of *q*_*n*_ are then modeled as a 1-D map (see also Eq (7)):

$$q_{n+1}=q_{n}+g\left(1+\sigma_{m}\nu_{m}\right)\;u_{q}\left(q_{n}\right)+\sigma_{a}\nu_{a},$$ (13)

where *q* is the controller state being regulated and *u*_*q*_(*q*_*n*_) is the control input to be derived. Here, *ν*_*m*_ and *ν*_*a*_ are each independent random variables with zero mean and unit variance that represent multiplicative (*ν*_*m*_) and additive (*ν*_*a*_) noise, respectively. The *σ*_*m*_ and *σ*_*a*_ terms then give the standard deviations of each noise term.

Similar to our prior work [9, 10], this state update equation (Eq (13)) models the discrete-time *inter*-step dynamics in *q*. As such, it represents a simple “control template” model that regulates noise-induced fluctuations away from perfect performance by adjusting *q*_*n*_ at each consecutive step. The choice of possible states to control (*q* ∈ {*z*_*B*_, Δ*z*_*B*_, *w*}; Fig 1) was motivated by the fact that any biped (human, robot, model, etc.) must generate some sequence of these variables to be “walking”. We note that in the absence of noise and control input, successive strides simply repeat (i.e., *q*_*n*+1_ = *q*_*n*_), which is consistent with well-established notions of limit cycle behavior (e.g., [16, 46, 75]) of walking. Thus, many reasonable models of continuous-time *within*-step walking dynamics, whether relatively very simple (e.g., [75, 76]), or highly complex (e.g., [78]) could be used to generate all of the relevant step-to-step time series (*z*_*Bn*_, Δ*z*_*Bn*_, *w*_*n*_, *z*_*Ln*_, *z*_*Rn*_) analyzed here.

Given Eqs (12) and (13), we then follow the same process as described in [9] to derive the control input, *u*_*q*_(*q*_*n*_). As in prior work [9, 10, 12], these controllers were modeled as stochastic optimal single-step controllers with direct error feedback, based on the Minimum Intervention Principle (MIP) [3, 4]. These controllers are optimal with respect to the expected value of the following cost function:

$$C=\alpha \;e_{n+1}^{2}+\gamma \;u_{q}^{2},$$ (14)

in which the first term penalized the error with respect to the goal function (Eq (12)) at the next step (i.e., the deviation of *F*_*q*_ from 0 at step *n* + 1) and the second term penalized “effort” in terms of the magnitude of the control input (*u*_*q*_) used to drive the state from step *n* to step *n*+1 [9]. Here, *α* and *γ* were positive constants that weighted the different components in the total cost, *C*. The form of Eq (14) is sufficiently general to include all cases studied in this work.

To derive *u*_*q*_(*q*_*n*_), we first substitute for *e* using *F*_*q*_ from Eq (12) into Eq (14):

$$C=\alpha \;{\left(q_{n+1}-q^{*}\right)}^{2}+\gamma \;u_{q}^{2}.$$ (15)

We then replace *q*_*n*+1_ with the right hand side of the state update map, Eq (13) to give:

C=α{[qn+(1+σmνm)uq+σaνa]−q*}2+γuq2. (16)

We next expand Eq (16) and gather like terms in $u_{q}^{2}$ and *u*_*q*_ to obtain:

$$\begin{array}{cc} C= & u_{q}^{2}\left(\alpha +2\alpha \sigma_{m}\nu_{m}+\alpha \sigma_{m}^{2}\nu_{m}^{2}+\gamma \right)+\ldots \\ & u_{q}\left(2\alpha \right)\left[\left(q_{n}-q^{*}\right)+\left(q_{n}-q^{*}\right)\sigma_{m}\nu_{m}+\sigma_{a}\nu_{a}+\sigma_{m}\sigma_{a}\nu_{m}\nu_{a}\right]+\ldots \\ & \alpha \left[{\left(q_{n}-q^{*}\right)}^{2}+2\left(q_{n}-q^{*}\right)\sigma_{a}\nu_{a}+\sigma_{a}^{2}\nu_{2}^{2}\right] \end{array}$$ (17)

We then take the expected value of C (i.e., $\bar{C}=E\left[C\right]$), noting that the noise processes have zero mean, unit variance, and are uncorrelated. That is, we set:

$$E\left[\nu_{m}^{2}\right]=E\left[\nu_{a}^{2}\right]=1$$

$$E\left[\nu_{m}\right]=E\left[\nu_{a}\right]=0$$

$$E\left[\nu_{m}\nu_{a}\right]=0$$

This then gives:

$$\bar{C}=E\left[C\right]=u_{q}^{2}\left(\alpha +\alpha \sigma_{m}^{2}+\gamma \right)+u_{q}\left[2\alpha \left(q_{n}-q^{*}\right)\right]+\alpha \left[{\left(q_{n}-q^{*}\right)}^{2}+\sigma_{a}^{2}\right].$$ (18)

In the general case, $\bar{C}$ defines the Lagrangian, Λ, for this system. The optimal controller then extremizes $\Lambda =\bar{C}$. We thus differentiate Eq (18) with respect to *u*_*q*_, set the resulting expression equal to zero, and solve the resulting algebraic equation for our final controller, *u*_*q*_ (see also Eq (8)):

$$\frac{\partial \bar{C}}{\partial u_{q}}=2\left(\alpha +\alpha \sigma_{m}^{2}+\gamma \right)u_{q}+2\alpha \left(q_{n}-q^{*}\right)=0$$ (19)

$$∴\;u_{q}\left(q_{n}\right)=-\left[\frac{1}{{\left(1+\sigma_{m}^{2}+\left(\gamma /\alpha \right)\right)}^{}}\right]\;\left(q_{n}-q^{*}\right),$$ (20)

where the term in brackets, [•], represents the effective gain of the controller that defines the amount of the error, (*q*_*n*_−q*), to be corrected at each step (see S1 Appendix for additional details). For each *q* ∈ {*z*_*B*_, Δ*z*_*B*_, *w*}, these models (Eqs (13) and (20)) were used to simulate the respective *q*_*n*_ time series. For each such simulated *q*_*n*_ time series, the relationships between each *q*_*n*_ and the other stepping variables (i.e., Eqs (2)–(4); Fig 1C) were then used to generate all of the relevant step-to-step time series (*z*_*Bn*_, Δ*z*_*Bn*_, *w*_*n*_, *z*_*Ln*_, *z*_*Rn*_) analyzed for each simulation.

Unlike our previous models for sagittal plane stepping regulation [9, 10], the controllers derived here (Eq (20)) were not “unbiased”, meaning they were not required to exhibit perfect performance *on average*. However, it remains relevant to derive the corresponding optimal control input for the more restrictive *un*biased case. Requiring *u*_*q*_(*q*_*n*_) to be unbiased would mean additionally requiring that the expected value of the goal function, *F*_*q*_, at step *n*+1 be zero [9]:

$$\bar{F}\equiv E\left[q_{n+1}-q^{*}\right]=0.$$ (21)

Substituting for *q*_*n*+1_ in *F* and taking expected values, this additional constraint becomes:

F¯≡E[qn+1−q*]=E[(qn−q*)+(1+σmνm)uq+σaνa]=(qn−q*)+uq=0 (22)

Adding this additional constraint to our controller yields the *augmented* Lagrangian, Λ, as:

$$\Lambda =\bar{C}-\mu \bar{F},$$ (23)

where *μ* is a Lagrange multiplier. The optimal controllers again extremize Λ. We thus make the appropriate substitutions in Eq (23), differentiate Λ, and set the resulting expression equal to zero to obtain:

$$\frac{\partial \bar{C}}{\partial u}-\mu \frac{\partial \bar{F}}{\partial u}=2\alpha \left(q_{n}-q^{*}\right)+2\left(\alpha +\alpha \sigma_{m}^{2}+\gamma \right)u_{q}-\mu =0.$$ (24)

Typically, Eqs (22) and (24) would be used together to solve for both *u*_*q*_ and *μ* [9, 12]. Here, as we are interested only in *u*_*q*_, we obtain the *un*biased stochastic optimal *u*_*q*_ directly from Eq (22) as:

$$u_{q}\left(q_{n}\right)=-\left(q_{n}-q^{*}\right).$$ (25)

Thus, this unbiased controller reduces to simple direct proportional feedback control. Alternatively, we could also obtain Eq (25) directly from Eq (20) in the special limiting case of assuming both no multiplicative noise and no cost for control effort (*σ*_*m*_ = *γ* = 0).

In particular, we note that for the case of *q* = *w*, Eq (25) is the same direct step width controller postulated by Kuo [16]. The present work thus extends that work in at least 3 important ways. First, all controllers considered here were *derived* from a stochastic optimal control framework, not proposed by conjecture. Second, these derived more general control policies (Eqs (8) and (11)) allow us to consider stochastically optimal controllers in cases less restrictive than the unbiased case. Third, they also allow us to consider stochastic control of other potentially relevant stepping variables beyond just step width.

### Multi-objective stochastic optimal regulator models

We consider strategies that control two controller states (*q*_1_ & *q*_2_) simultaneously, with the goal to drive each variable to its own desired value. Thus, the generic dual-objective goal function has the form of Eq (9), with the expanded two-state update map of Eq (10). We then take the cost function for regulating two variables to be the sum of those used for each single variable:

$$C=\alpha_{1}e_{1,n+1}^{2}+\gamma_{1}u_{q_{1}}^{2}+\alpha_{2}e_{2,n+1}^{2}+\gamma_{2}u_{q_{2}}^{2}.$$ (26)

The subsequent steps in the derivation are then the same as those described above for the single variable case, as outlined in Eqs (15)–(20). Here, this yields the two-variable control law of Eq (11). We considered three different specific versions of this general model, corresponding to all the possible combinations of any two of the three potential control state variables: i.e., [*q*_1_, *q*_2_] ∈ {[*z*_*B*_, Δ*z*_*B*_], [Δ*z*_*B*_, *w*], [*z*_*B*_, *w*]}.

We note that the goal function of Eq (9) indicates that each error, *e*_*i*_, in Eq (26) will depend only on the goal level error for its own corresponding control state variable, *q*_*i*_. Likewise, Eq (26) contains no cross terms in either the goal-level errors (*e*_1_, *e*_2_) or controller inputs (*u*_1_, *u*_2_). Hence, the final derived control law of Eq (11) and corresponding final state update equation of Eq (10) are both fully diagonal, such that the fluctuation dynamics of *q*_1_ and *q*_2_ are uncoupled. While it is certainly possible to imagine controller templates that include interactions between *q*_1_ and *q*_2_, we found in practice that the more parsimonious forms of Eqs (9) and (26) were adequate for our analyses.

### Simulations of lateral stepping

In total, we generated simulations for three sets of each of three models. We first assessed the original (unconstrained) uni-objective models for each of *q* ∈ {*z*_*B*_, Δ*z*_*B*_, *w*}, for which we imposed no additional task constraints. We assessed each control strategy across a range of parameter values of each model. We then compared the dynamical behavior predicted by each set of simulations directly to experimental values (i.e., Fig 2) to determine the likelihood humans might have adopted any of these uni-objective control strategies. Full details of these simulations and the parameter sensitivity analyses conducted on these models and the results of those analyses are in S1 Appendix.

All of the unconstrained uni-objective models failed to capture human stepping dynamics (S1 Appendix). They did so mainly by either exceeding lateral boundary limits (i.e., they walked off the path) and/or by taking steps that were unrealistically too wide or too narrow.

Because none of the *un*constrained uni-objective models captured human stepping dynamics, we then assessed corresponding “constrained” uni-objective models for each of *q* ∈ {*z*_*B*_, Δ*z*_*B*_, *w*}. In those simulations we additionally imposed physiologically relevant constraints that the simulations could not take steps that would walk off the path or would yield step widths unrealistically too wide or too narrow. We assessed each of these constrained control strategies across a range of parameter values of each model. We then compared the dynamical behavior predicted by each set of these simulations directly to human experimental values (i.e., Fig 2). Full details of these simulations and the parameter sensitivity analyses conducted on these models and the results of those analyses are in S1 Appendix.

Finally, we assessed each of the three 2-variable multi-objective models (i.e., [*q*_1_, *q*_2_] ∈ {[*z*_*B*_, Δ*z*_*B*_], [Δ*z*_*B*_, *w*], [*z*_*B*_, *w*]}). We assessed each of these control strategies across a range of parameter values of each model. We then compared the dynamical behavior predicted by each set of these simulations directly to experimental values (i.e., Fig 2) to determine the likelihood humans might have adopted any of these multi-objective control strategies. Full details of these simulations and the parameter sensitivity analyses conducted on these models and the results of those analyses are in S2 Appendix.

### Calculation of dependent measures

The primary gait variables obtained from each simulated or experimental walking trial consisted of time series of left and right lateral foot placements (*z*_*L*_ & *z*_*R*_), lateral body position and heading (*z*_*B*_ & Δ*z*_*B*_), and step width (*w*) (Figs 1C and 2). We then analyzed these times series from both experimental and simulated walking trials in the same ways for each to make direct comparisons between simulation predictions and experimental findings.

First, we computed standard deviations of each relevant time series to quantify the magnitudes of the variance in them. For the computational models, variance in the respective variable(s) being regulated reflected the “goal relevant” variance [6, 7] because fluctuations in each respective controlled *q*_*n*_ directly reflected deviations away from the corresponding desired q*. Conversely, any variance in those variables not being directly controlled was “goal equivalent” [6, 7] because fluctuations in those variables did not directly affect the designated task goal(s).

Standard deviations, however, quantify only the average magnitude of differences across all steps, regardless of temporal order. They thus yield *no* information about how each step affects subsequent steps. Therefore, here we used Detrended Fluctuation Analysis (DFA) to define a convenient lag-independent measure of statistical persistence across successive steps in each time series [9, 12, 71]. In brief, DFA calculates a scaling exponent, *α*, that quantifies the degree of statistical persistence or anti-persistence in a time series. To compute these exponents, each time series *x*(*n*), where *n* ∈ {1, …, *N* } steps [3–5], was first integrated to form a cumulative sum:

$$y\left(k\right)=\sum_{i=1}^{k}\left[x\left(n\right)-\bar{x}\right]$$ (27)

where *x*(*n*) is the value of *x* for the *n*^th^ step and $\bar{x}$ is the mean value of *x* across all *N* strides. Each integrated series was divided into equal, non-overlapping segments of length *j*. Each segment was detrended by subtracting a least squares linear fit to that segment. The squares of the residuals were then averaged over the entire data set and the square root of the mean residual, *F*(*j*), was calculated:

$$F(j)=\sqrt{\frac{1}{N}\sum_{k=1}^{N}{\left[y(k)-y_{fit}(k)\right]}^{2}}$$ (28)

This process was repeated for different segment lengths, *j*. Here, fifty values of *j* evenly distributed between 4 and *N*/4 were used. Typically, *F*(*j*) increases with *j* and a graph of log[*F*(*j*)] versus log(*j*) will exhibit a power-law relationship indicating the presence of scaling, such that *F*(*j*) ≈ *j*^*α*^. These log[*F*(*j*)] versus log(*j*) plots were then fit with a linear function using least squares regression. The slope of this line defined the scaling exponent α [9, 12, 71].

Standard deviation calculations quantify only average *magnitudes* of fluctuations and DFA α calculations quantify only the degree to which, on average, these deviations are corrected in *time* (from step-to-step). Neither analysis quantifies how step-to-step corrections of fluctuations might depend nonlinearly on the initial magnitudes of those fluctuations. Thus here, we directly quantified the degree to which deviations, ${q^{\prime }}_{n}=q_{n}-\bar{q}$, from the mean value,$\bar{q}$, of a given time series were corrected on the subsequent step by corresponding changes, Δ*q*_n+1_ = *q*_n+1_ − *q*_n_, in the opposite direction [10]. We performed these analyses on each relevant time series obtained from both our human subjects (Fig 2) and from each of our final multi-objective position-step width control models (Fig 5). For each relevant time series, *q* ∈ {*z*_*B*_, Δ*z*_*B*_, *w*}, we constructed plots of Δ*q*_n+1_ vs. *q'*_n_ (Fig 6A). We then computed the linear slopes (using least-squares regression) and strength of correlation (r^2^) [10] for each corresponding relationship (Fig 6B and 6C).

### Statistical comparisons

The primary comparisons of interest were between the model predictions and experimental observations (Fig 2). Here however, because the simulated and experimental data were structured in very different ways, standard inferential statistical tests (e.g., t-test, ANOVA, etc.) would not be appropriate. Additionally, because for the models, we could simulate as many trials as we wanted and of any arbitrary length, we could thus ensure p-values for most any comparisons that could be arbitrarily small and thus would not be meaningful. Therefore, to present descriptive data (Figs 2, 3, 4 & 7), for each relevant measure, data were plotted as boxplots indicating the median, 1^st^ and 3^rd^ quartiles, with whiskers extending to 1.5× the inter-quartile range and any values beyond that range indicated by individual data points. To directly compare model predictions to human experimental results (Figs 5 and 6), for each relevant measure, the expected *range* of values exhibited by humans was taken as the mean ± 1 standard deviation for the experimental data set. Model predictions are then shown as the mean ± 95% confidence interval for that mean for each set of corresponding simulations. We then infer that whenever the mean simulation prediction for a given measure lies within the experimental mean ± 1 SD band, that computational prediction is statistically consistent with experimental findings [10].

### Data

All relevant experimental data are available from Dryad (https://doi.org/10.5061/dryad.p254480) [83].

## Supporting information

S1 Appendix Uni‐objective models: Results of parameter sensitivity analyses. (PDF)

S2 Appendix Multi‐objective models: Results of parameter sensitivity analyses. (PDF)
