# Supplementary material for: Humans use multi-objective control to regulate lateral foot placement when walking
Source: PLoS Comput Biol. 2019 Mar 6;15(3):e1006850. doi: 10.1371/journal.pcbi.1006850 (PMC6422313; doi:10.1371/journal.pcbi.1006850)
Supplement: S1 Appendix — (PDF) [file pcbi.1006850.s001.pdf]

HUMANS USE MULTI-OBJECTIVE CONTROL TO REGULATE LATERAL FOOT PLACEMENT WHEN WALKING

Jonathan B. Dingwell and Joseph P. Cusumano

PLoS Computational Biology

SUPPLEMENTARY TEXT #S1
Uni-Objective Models: Results of Parameter Sensitivity Analyses

---

To determine the likelihood humans adopt any given uni-objective control strategy (i.e., regulating any of lateral position (z_B), heading (Δz_B), or step width (w) individually), we conducted parameter sensitivity studies of each uni-objective model. We compared the results of model outputs directly to human experimental data (Fig. 2).

We first note that each model was governed by the state update equation (Eq. 7) and feedback control law (Eq. 8), such that its behavior was dictated by 3 parameters: additive noise amplitude (σ_a), multiplicative noise amplitude (σ_m), and the relative weights (error correction (α) vs. control effort (γ)) from the cost function (γ/α). We further note that not only did redundancy exist between the output variables examined (z_B, Δz_B, and w), but redundancy also existed *within the control law* itself: i.e., infinite combinations of σ_m and γ/α could yield the exact same effective controller gain:

$$q_{n+1} = q_n + g\left(1+\sigma_m \nu_m\right)u_q + \sigma_a \nu_a \qquad \text{Eq. (7)}$$

$$u_q\left(q_n\right) = -\left[\frac{1}{\left(1+\sigma_m^2+\left(\gamma/\alpha\right)\right)}\right]\left(q_n - q^*\right) \qquad \text{Eq. (8)}$$

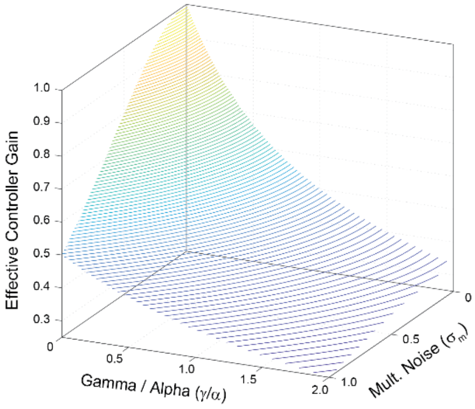

Right: Iso-gain contour plot for the effective controller gain, [•] from Eq. (8), as a function of amplitude of multiplicative noise (σ_m) and ratio of the cost function weights (γ/α). Each horizontal line represents the set of all combinations of σ_m and γ/α that yield the exact same effective gain for the controller input: $u_q(q_n)$.

We systematically varied each of these parameters (σ_a, σ_m, γ/α) one-at-a-time to determine their effects on the model outputs. For each variable regulated (z_B, Δz_B, w), we defined the 'baseline' value for additive noise (σ'_a) for that variable as the group mean of the within-subject standard deviation for that variable, based on the experimental data (Fig. 2):

	Position (z_B)	Heading (Δz_B)	Step Width (w)
Baseline Additive Noise (σ'_a):	0.0424 m	0.0160 m	0.0257 m

We took the baseline amplitude of multiplicative noise (σ'_m) to be 10% of the baseline additive noise (i.e., $\sigma'_m = 0.10\cdot\sigma'_a$), as multiplicative noise effects are quite small for steady-state walking [1, 2]. We also presumed error correction was weighted more heavily than control effort, and thus we took the baseline ratio of the cost function weights to be $(\gamma/\alpha)' = 0.10$.

Each parameter to be varied was then varied over a range of values much wider than expected to be observed empirically. Additive noise amplitudes (σ_a) were varied from 0 to 5 times σ'_a. Multiplicative noise amplitudes (σ_m) were varied from 0 to 20 times σ'_a (i.e., 200 times 'baseline'). Relative cost function weights (γ/α) were varied from 0 to 2 (i.e., up to *twice* as much weight placed on control effort than error correction). These parameter variations are summarized as follows:

	To Vary σ_a:	To Vary σ_m:	To Vary γ/α:
σ_a:	Vary: $\{0, \ldots, (5\cdot\sigma'_a)\}$	Fixed: σ'_a	Fixed: σ'_a
σ_m:	Fixed: $0.10\cdot\sigma'_a$	Vary: $\{0, \ldots, (20\cdot\sigma'_a)\}$	Fixed: $0.10\cdot\sigma'_a$
γ/α:	Fixed: 0.10	Fixed: 0.10	Vary: $\{0, \ldots, 2\}$
Plot Color:	Green	Red	Blue

The "Normalized Parameter Range [a.u]" shown as {0, …, 1} on the horizontal axes in each of the following "Parameter Sensitivity Results" figures (Figs. S1-2, S1-4, S1-6, S1-8, S1-10, and S1-12) corresponds to the ranges listed above.

We took the 'desired' or 'goal' values for each variable to be:

Position: $z^*_B = 0.0$ m (center of path)	Heading: $\Delta z^*_B = 0.0$ m (straight ahead)	Step Width: $(w^*) = 0.127$ m (from experiment – Fig. 2)

Where the desired step width (w^*) was the group mean of the within-subject mean step width from the experiment (Fig. 2).

For each model and each set of parameter values tested, we generated 20 sets of time series data, each simulating 500 consecutive walking steps (analogous to simulating 20 walking trials). Time series of left & right foot positions (z_L and z_R), body position (z_B), heading (Δz_B), and step width (w) were generated for each simulated walking trial and analyzed in the same manner as experimental data: standard deviations (σ) and DFA exponents (α) were computed for each time series.

Beyond the control inputs as specified by the model, these initial models were otherwise "unconstrained" and thus capable of making stepping movements that could step off the path, or with very wide or very narrow step widths. We therefore also computed the percentage of steps (z_L and z_R) taken in each simulated trial that exceeded the *Lateral Boundary Limits*: i.e., for the experiments analyzed here, the left and right edges of the treadmill belt (±0.885 m, see Fig. 2).

We also computed the percentage of steps that exhibited step widths that we defined as unrealistically either "too wide" or "too narrow". We defined this as exceeding ±5 standard deviations (±5σ), as determined from the group-average of the within-subject standard deviations, averaged across all participants (Fig. 2). This yielded an allowable step width range of −0.15 cm to +25.54 cm (see Fig. 2). Statistically, ±5σ encompasses >>99.99% of the observed data, reflecting an ~1 in 3.5 million chance of stepping outside this range. However, when asked to do so, healthy adults can walk with steps as wide as ~29 to 42 cm [3][4][5] and can take crossover steps [6-8] with step widths well below the −0.15 cm. Thus, while exceeding these ±5σ limits would be very unusual for normal walking, doing so would still be very biomechanically feasible.

Imposing Realistic Biomechanical Constraints:

All of the initially "unconstrained" models (Figs. S1-1 thru S1-6) failed to adequately capture the stepping dynamics exhibited by humans (Fig. 2), regardless of parameter choices. In most cases, these models either regularly exceeded either the lateral boundary limits (i.e., they stepped off the treadmill) or the ±5σ step width limits, or both.

We therefore ran additional simulations and repeated the same parameter sensitivity analyses (Figs. S1-7 thru S1-12) where this time we retained each of the same three controllers, but imposed constraints on foot placement (z_L and z_R) at each step such that the simulations could not step off the treadmill (±0.885 m) and could not take steps that would exceed the ±5σ step width limits (i.e., −0.15 to +25.54 cm) described above.

It is important to note that all of these "constrained" uni-objective control models both fully satisfied the requirements of the task (i.e., Eq. 1) and did so by taking steps that were biomechanically feasible [3-8]. That is, *all* of these simulations reflected stepping strategies people *could* have executed successfully, had they chosen to.

It is also important to note that the constraints imposed were intended to reflect those minimally necessary to achieve the task goal (Eq. 1). Had we imposed constraints on foot placement more (or less) restrictive than those imposed here, neither the overall qualitative findings, nor the final conclusions, would have been any different.

Figures and Plotting Conventions:

For each of the six total control models, we present two figures. In the first figure for each model (odd numbered figures), we display example time series and comparisons to human values for 'baseline' parameter values. For each output variable, we show comparisons of boxplots to demonstrate the range and distribution of values within each data set.

For each second (even numbered) figure, we show the mean ± 1σ bands for humans (gray shaded areas) to indicate the *range* of values observed experimentally for that variable. We plot simulation results as mean ±95% confidence intervals for each mean to identify where the predicted simulation means would lie inside or outside of the experimental range.

Position Control:

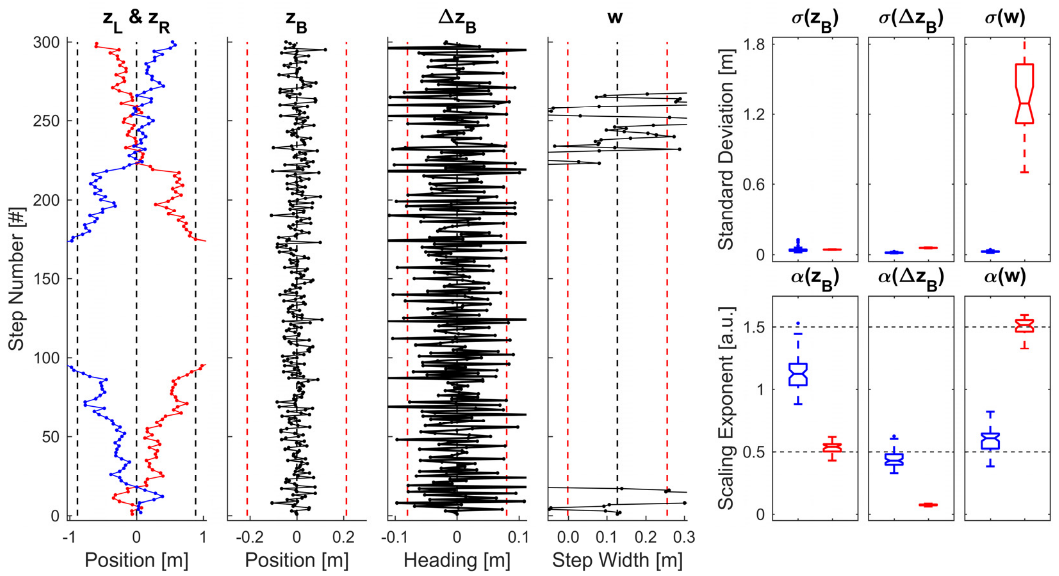

Figure S1-1: Example Results. Left: Typical time series of left & right foot placements (z_L & z_R), body position (z_B), heading (Δz_B), and step width (w) for baseline parameter values (see text). Right: Box plots of variability (standard deviations) and DFA α results for each variable for the baseline model (red: 30 simulated trials of 500 steps each) and humans (blue: 65 total trials of 290 steps each from 13 participants (5 trials each)).

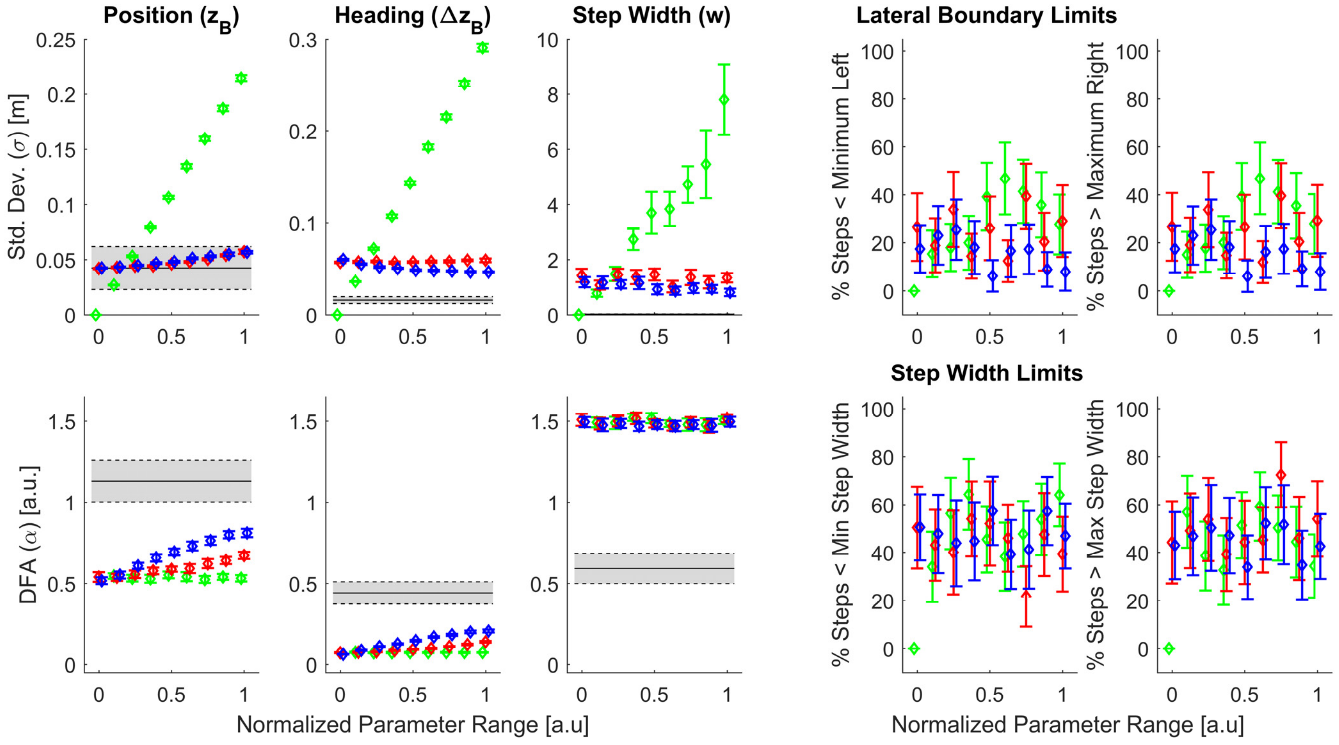

Figure S1-2: Parameter Sensitivity Results. Each set of point represents variations in additive noise (σ_A: Green), multiplicative noise (σ_M: Red), or relative cost function gains (γ/α: Blue) over the range of values tested for each (see text). Left: Variability and DFA α results. Error bars indicate ±95%CI for the mean of each set of simulations. Gray horizontal bands indicate the range (mean±SD) exhibited by humans in the experiment. Right: Percentage of steps across trials that exceeded either lateral boundary or step width limits. Error bars indicate ±95%CI for each mean.

<u>Heading Control:</u>

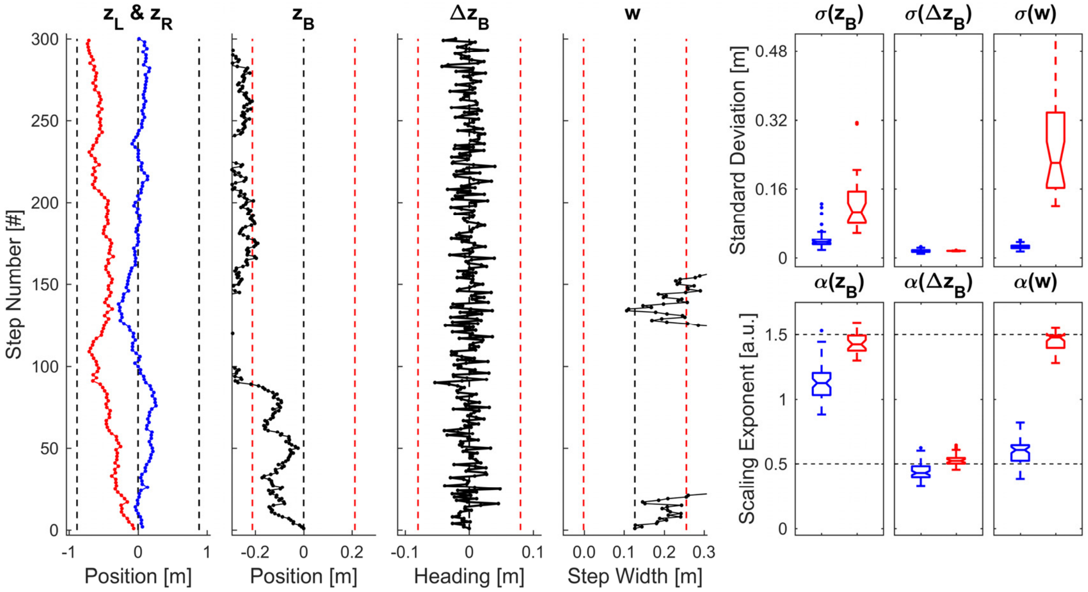

Figure S1-3: Example Results. Left: Typical time series of left & right foot placements (z_L & z_R), body position (z_B), heading (Δz_B), and step width (w) for baseline parameter values (see text). Right: Box plots of variability (standard deviations) and DFA α results for each variable for the baseline model (red: 30 simulated trials of 500 steps each) and humans (blue: 65 total trials of 290 steps each from 13 participants (5 trials each)).

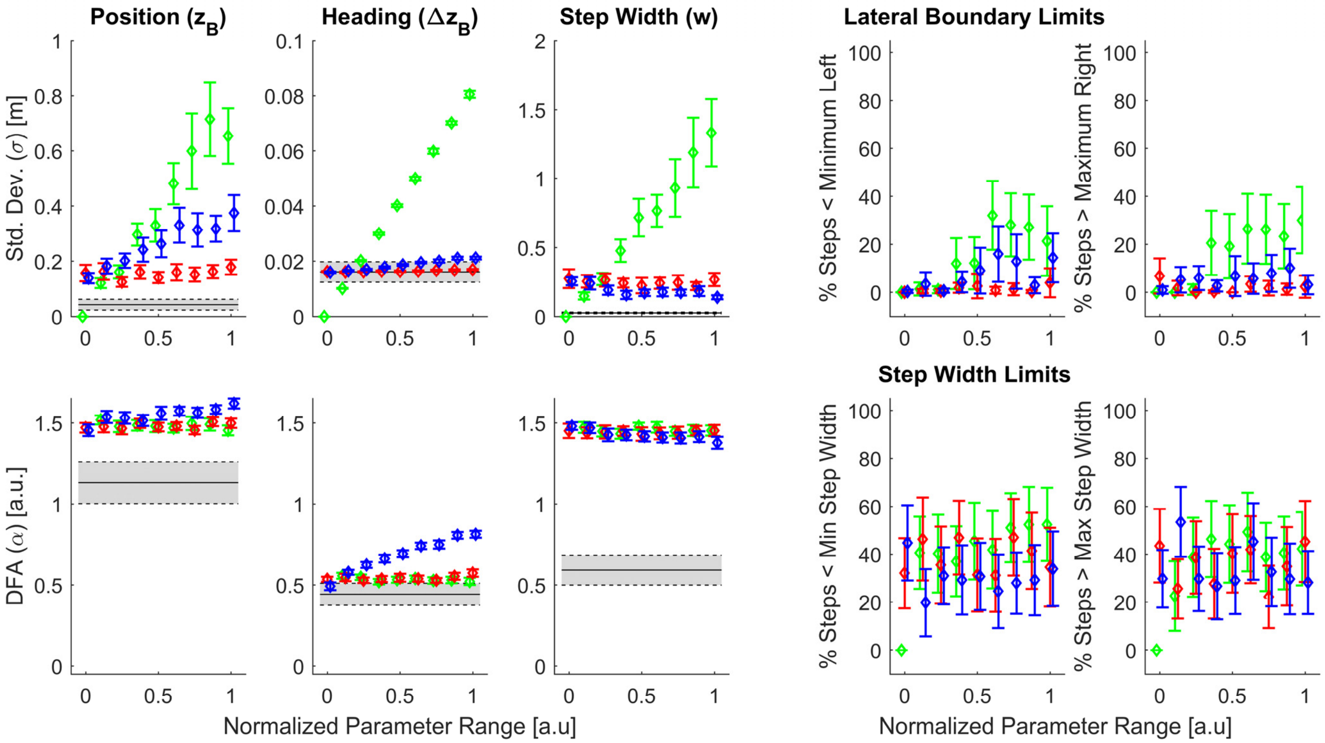

Figure S1-4: Parameter Sensitivity Results. Each set of point represents variations in additive noise (σ_A: Green), multiplicative noise (σ_M: Red), or relative cost function gains (γ/α: Blue) over the range of values tested for each (see text). Left: Variability and DFA α results. Error bars indicate ±95%CI for the mean of each set of simulations. Gray horizontal bands indicate the range (mean±SD) exhibited by humans in the experiment. Right: Percentage of steps across trials that exceeded either lateral boundary or step width limits. Error bars indicate ±95%CI for each mean.

Step Width Control:

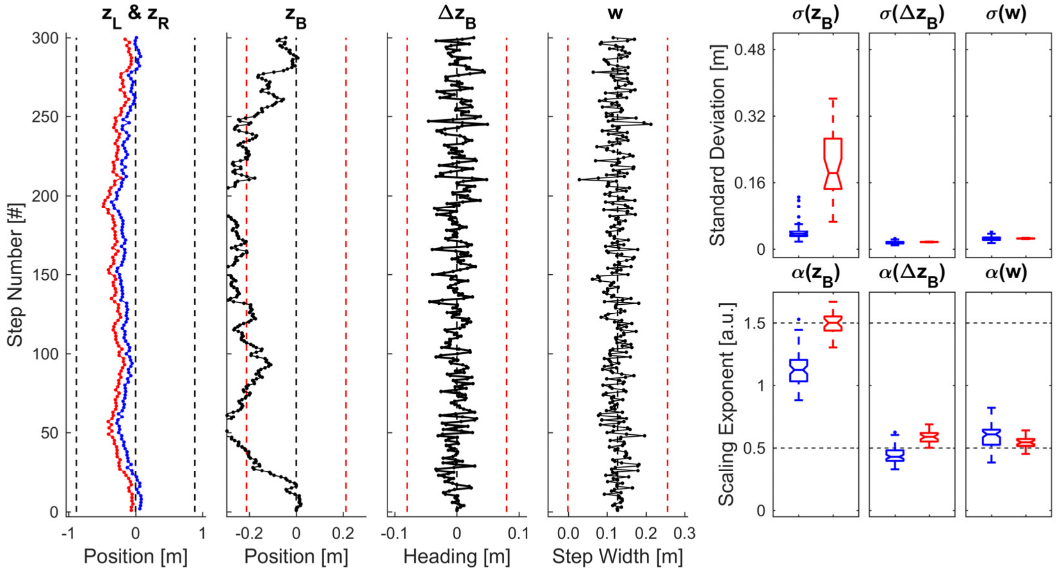

Figure S1-5: Example Results. Left: Typical time series of left & right foot placements (z_L & z_R), body position (z_B), heading (Δz_B), and step width (w) for baseline parameter values (see text). Right: Box plots of variability (standard deviations) and DFA α results for each variable for the baseline model (red: 30 simulated trials of 500 steps each) and humans (blue: 65 total trials of 290 steps each from 13 participants (5 trials each)).

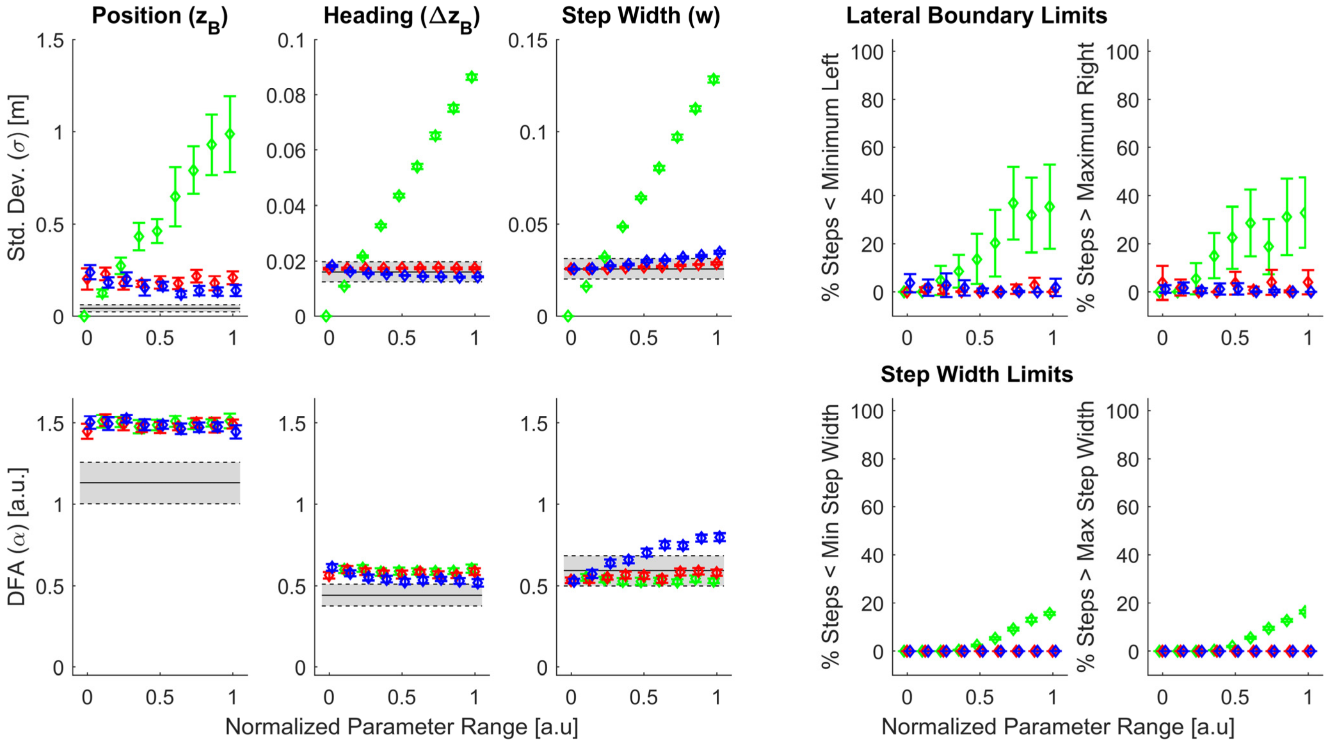

Figure S1-6: Parameter Sensitivity Results. Each set of point represents variations in additive noise (σ_A: Green), multiplicative noise (σ_M: Red), or relative cost function gains (γ/α: Blue) over the range of values tested for each (see text). Left: Variability and DFA α results. Error bars indicate ±95%CI for the mean of each set of simulations. Gray horizontal bands indicate the range (mean±SD) exhibited by humans in the experiment. Right: Percentage of steps across trials that exceeded either lateral boundary or step width limits. Error bars indicate ±95%CI for each mean.

<u>Position Control – Constrained:</u>

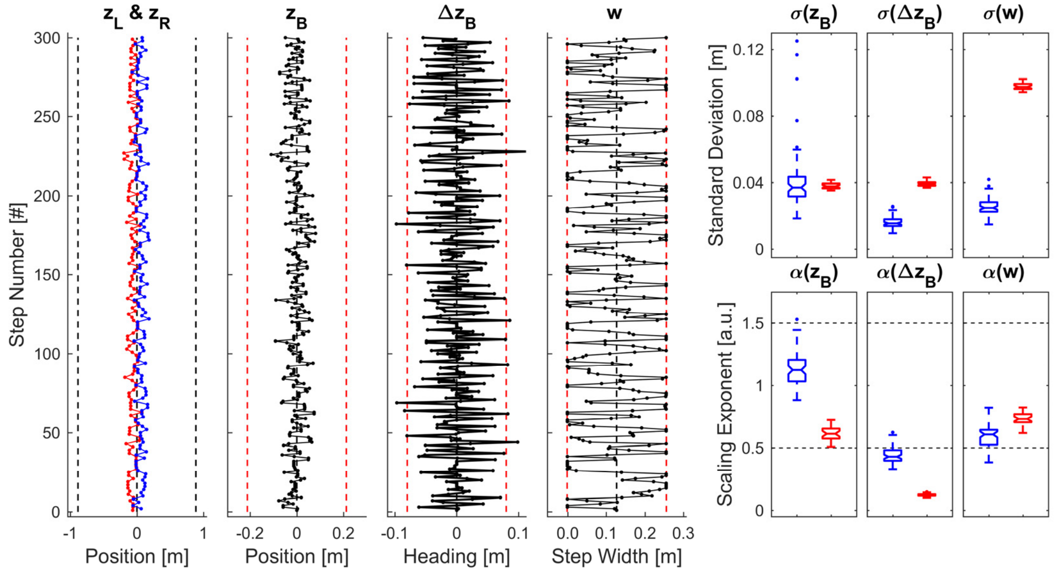

Figure S1-7: Example Results. Left: Typical time series of left & right foot placements (z_L & z_R), body position (z_B), heading (Δz_B), and step width (w) for baseline parameter values (see text). Right: Box plots of variability (standard deviations) and DFA α results for each variable for the baseline model (red: 30 simulated trials of 500 steps each) and humans (blue: 65 total trials of 290 steps each from 13 participants (5 trials each)).

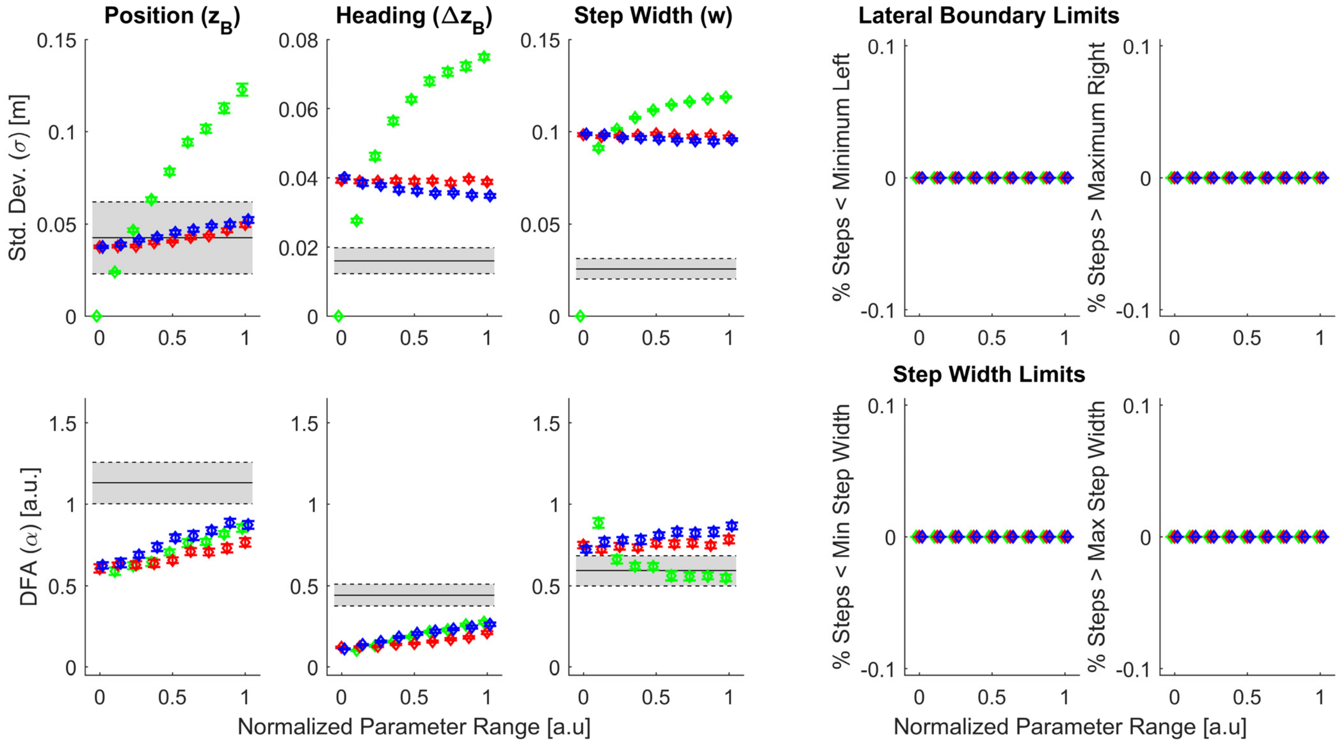

Figure S1-8: Parameter Sensitivity Results. Each set of point represents variations in additive noise (σ_A: Green), multiplicative noise (σ_M: Red), or relative cost function gains (γ/α: Blue) over the range of values tested for each (see text). Left: Variability and DFA α results. Error bars indicate ±95%CI for the mean of each set of simulations. Gray horizontal bands indicate the range (mean±SD) exhibited by humans in the experiment. Right: Percentage of steps across trials that exceeded either lateral boundary or step width limits. Error bars indicate ±95%CI for each mean.

Heading Control – Constrained:

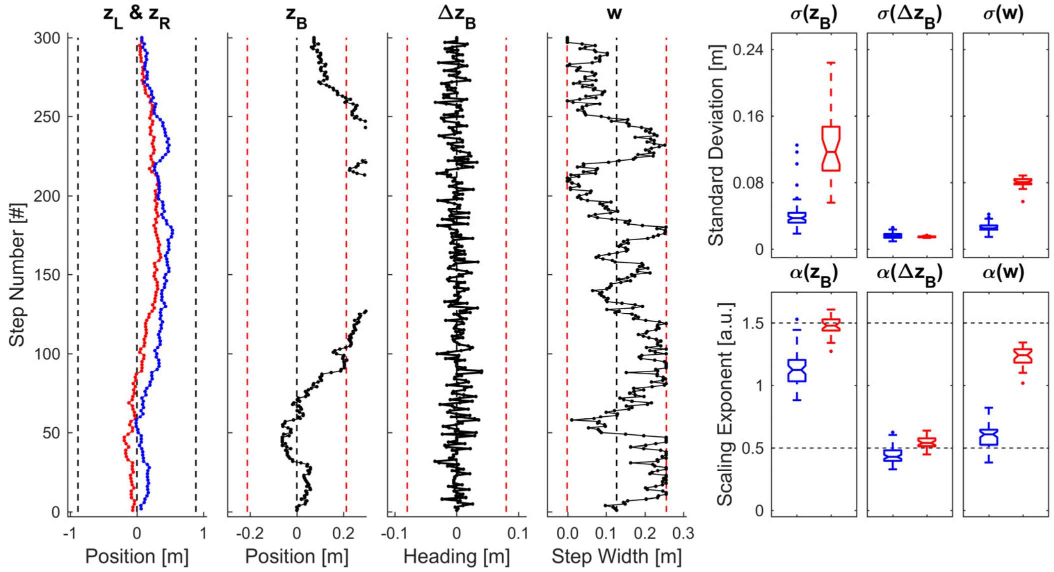

Figure S1-9: Example Results. Left: Typical time series of left & right foot placements (z_L & z_R), body position (z_B), heading (Δz_B), and step width (w) for baseline parameter values (see text). Right: Box plots of variability (standard deviations) and DFA α results for each variable for the baseline model (red: 30 simulated trials of 500 steps each) and humans (blue: 65 total trials of 290 steps each from 13 participants (5 trials each)).

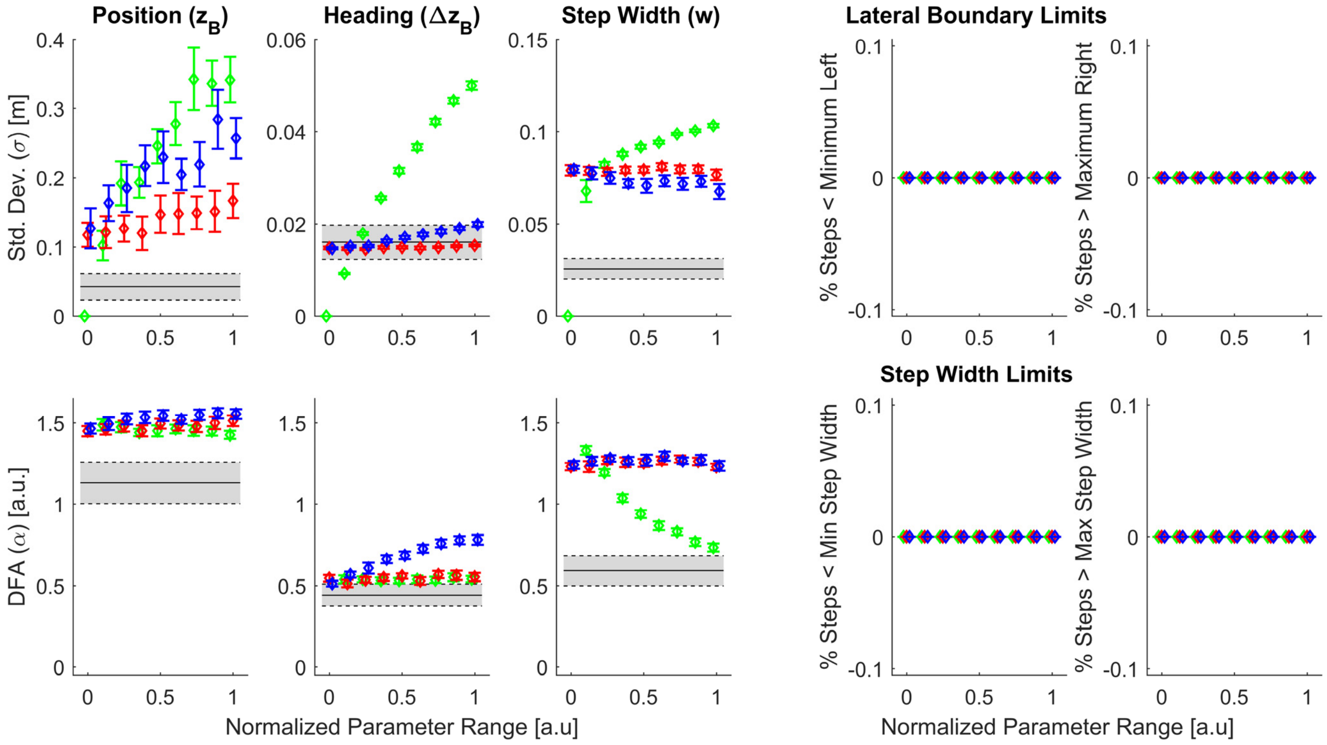

Figure S1-10: Parameter Sensitivity Results. Each set of point represents variations in additive noise (σ_A: Green), multiplicative noise (σ_M: Red), or relative cost function gains (γ/α: Blue) over the range of values tested for each (see text). Left: Variability and DFA α results. Error bars indicate ±95%CI for the mean of each set of simulations. Gray horizontal bands indicate the range (mean±SD) exhibited by humans in the experiment. Right: Percentage of steps across trials that exceeded either lateral boundary or step width limits. Error bars indicate ±95%CI for each mean.

Step Width Control – Constrained:

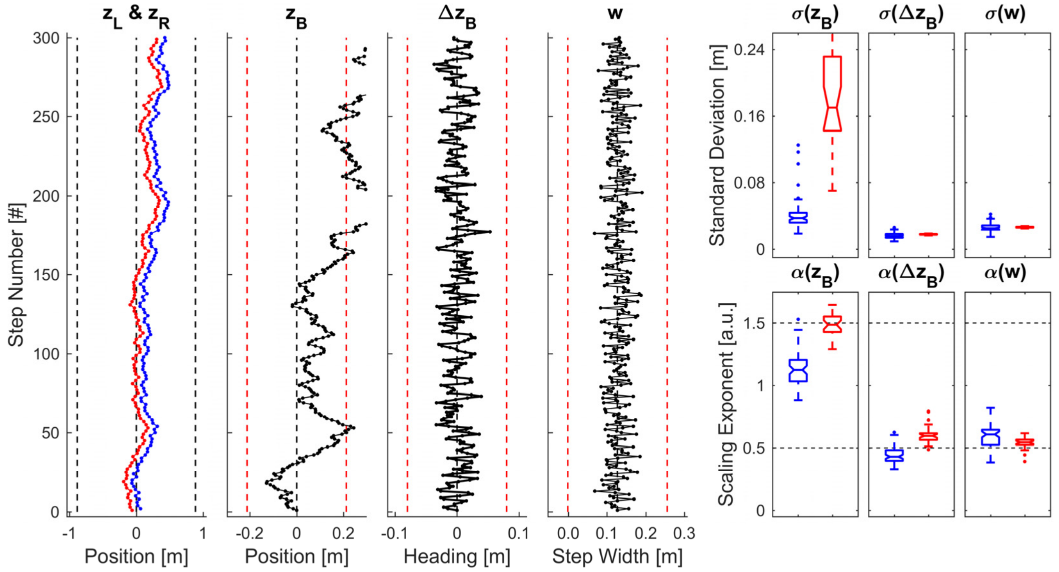

Figure S1-11: Example Results. Left: Typical time series of left & right foot placements (z_L & z_R), body position (z_B), heading (Δz_B), and step width (w) for baseline parameter values (see text). Right: Box plots of variability (standard deviations) and DFA α results for each variable for the baseline model (red: 30 simulated trials of 500 steps each) and humans (blue: 65 total trials of 290 steps each from 13 participants (5 trials each)).

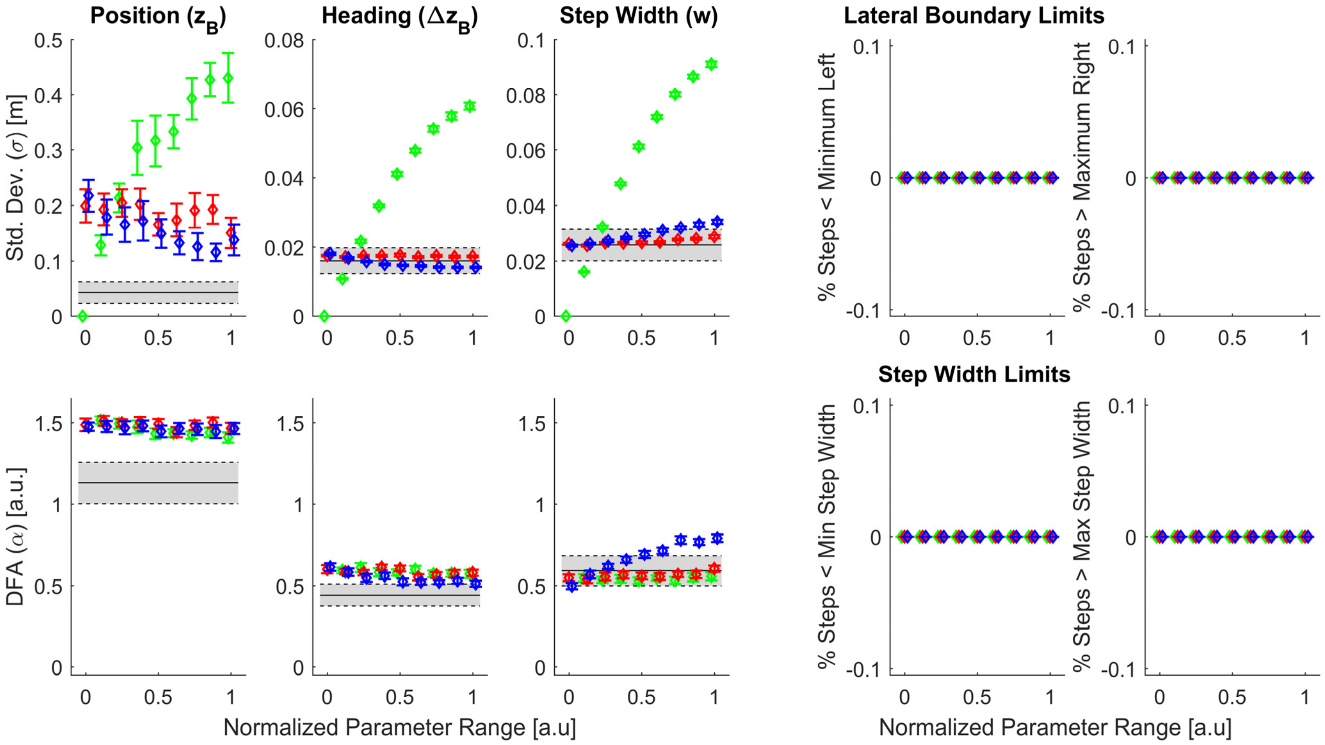

Figure S1-12: Parameter Sensitivity Results. Each set of point represents variations in additive noise (σ_A: Green), multiplicative noise (σ_M: Red), or relative cost function gains (γ/α: Blue) over the range of values tested for each (see text). Left: Variability and DFA α results. Error bars indicate ±95%CI for the mean of each set of simulations. Gray horizontal bands indicate the range (mean±SD) exhibited by humans in the experiment. Right: Percentage of steps across trials that exceeded either lateral boundary or step width limits. Error bars indicate ±95%CI for each mean.

Summary / Conclusions:

Unconstrained Uni-Objective Control Models (Figs. S1-1 to S1-6):

Comparisons across models exhibit clear redundancy between the output variables (z_B, Δz_B, and w): when any one variable was regulated, the others clearly were not. All model configurations were highly sensitive to additive noise (σ_a), highly *in*sensitive to even very large changes in multiplicative noise (σ_m), and only modestly sensitive to changes in relative cost function weights (γ/α).

All of these models failed to capture the stepping dynamics exhibited by humans (Fig. 2) across all parameter combinations tested. In addition to not matching variability and/or DFA results, most simulations regularly exceeded either lateral boundary or step width limits, or both. In particular, while the DFA results were highly sensitive (between models) to which stepping variable was regulated, they were highly *in*sensitive to any of the imposed variations in the parameters governing each model. Thus, control of z_B (Fig. S1-2) always resulted in $\alpha(w) \approx 1.5$, control of Δz_B always resulted in $\alpha(z_B) \approx \alpha(w) \approx 1.5$, and control of w (Fig. S1-6) always resulted in $\alpha(z_B) \approx 1.5$. In each case, the DFA $\alpha \approx 1.5$ reflected an uncontrolled random walk process [9, 10]. The failure of these models to replicate human stepping dynamics was thus not due to some limitation in the choice of parameters, but rather to fundamental structural differences in the models themselves.

Constrained Uni-Objective Control Models (Figs. S1-7 to S1-12):

Adding relevant constraints to choices of stepping locations (z_L & z_R) ensured that *ALL* of these simulations solved the main task requirement (Eq. 1) without ever stepping off of the treadmill or taking biomechanically implausible steps (% of steps exceeding lateral boundary or step width limits all = 0). This mitigated some of the excessive variability observed in some of the unconstrained control models and thus some values of some stepping variables improved for some parameter choices.

However, all of these constrained models still failed to capture the stepping dynamics exhibited by humans (Fig. 2) across *all* of the parameter choices tested. Thus, in spite of the fact that *all* of these models produced biomechanically viable strategies, none of them came *close* to replicating what we had observed in the experimental findings.

References:

1. Dingwell JB, Salinas MM, Cusumano JP. Increased Gait Variability May Not Imply Impaired Stride-To-Stride Control of Walking in Healthy Older Adults. Gait Posture. 2017;55:131-7. doi: https://doi.org/10.1016/j.gaitpost.2017.03.018.
2. Dingwell JB, Cusumano JP. Identifying Stride-To-Stride Control Strategies in Human Treadmill Walking. PLoS ONE. 2015;10(4):e0124879. doi: http://dx.doi.org/10.1371/journal.pone.0124879.
3. McAndrew Young PM, Dingwell JB. Voluntarily changing step length or step width affects dynamic stability of human walking. Gait Posture. 2012;35(3):472-7. doi: http://dx.doi.org/10.1016/j.gaitpost.2011.11.010.
4. Perry JA, Srinivasan M. Walking with wider steps changes foot placement control, increases kinematic variability and does not improve linear stability. Royal Society Open Science. 2017;4(9). doi: https://doi.org/10.1098/rsos.160627.
5. Donelan JM, Kram R, Kuo AD. Mechanical and metabolic determinants of the preferred step width in human walking. Proc Roy Soc B Biol Sci. 2001;268(1480):1985-92. doi: https://doi.org/10.1098/rspb.2001.1761.
6. Thies SB, Ashton-Miller JA, Richardson JK. What causes a crossover step when walking on uneven ground?: A study in healthy young women. Gait Posture. 2007;26(1):156-60. doi: http://dx.doi.org/10.1016/j.gaitpost.2006.08.011.
7. Hilliard MJ, Martinez KM, Janssen I, Edwards B, Mille M-L, Zhang Y, et al. Lateral Balance Factors Predict Future Falls in Community-Living Older Adults. Arch Phys Med Rehabil. 2008;89(9):1708-13. doi: http://dx.doi.org/10.1016/j.apmr.2008.01.023.
8. Hurt CP, Grabiner MD. Age-related differences in the maintenance of frontal plane dynamic stability while stepping to targets. J Biomech. 2015;48(4):592-7. doi: https://doi.org/10.1016/j.jbiomech.2015.01.003.
9. John J, Dingwell JB, Cusumano JP. Error Correction and the Structure of Inter-Trial Fluctuations in a Redundant Movement Task. PLoS Comput Biol. 2016;12(9):e1005118. doi: http://dx.doi.org/10.1371/journal.pcbi.1005118.
10. Dingwell JB, Cusumano JP. Re-Interpreting Detrended Fluctuation Analyses of Stride-To-Stride Variability in Human Walking. Gait Posture. 2010;32(3):348-53. doi: http://dx.doi.org/10.1016/j.gaitpost.2010.06.004.
