# Supplementary material for: Humans use multi-objective control to regulate lateral foot placement when walking
Source: PLoS Comput Biol. 2019 Mar 6;15(3):e1006850. doi: 10.1371/journal.pcbi.1006850 (PMC6422313; doi:10.1371/journal.pcbi.1006850)
Supplement: S2 Appendix — (PDF) [file pcbi.1006850.s002.pdf]

Humans Use Multi-Objective Control to Regulate Lateral Foot Placement When Walking

Jonathan B. Dingwell and Joseph P. Cusumano
PLoS Computational Biology

SUPPLEMENTARY TEXT #S2
Multi-Objective Models: Results of Parameter Sensitivity Analyses

To determine the likelihood humans use any given *multi*-objective control strategy, i.e., regulating any combination of $[q_1, q_2] \in \{[z_B, \Delta z_B], [\Delta z_B, w], [z_B, w]\}$, we conducted parameter sensitivity studies of each multi-objective model. We compared results of model outputs directly to human experimental data (Fig. 2).

For each control state variable combination, we systematically varied the proportion (ρ) of control between the two variables (q_1 and q_2) being regulated, such that ρ = 0.0 reflected 100% q_1 control and ρ = 1.0 reflected 100% q_2 control. The "Multi-Objective Proportion" range shown as {0, …, 1} on the horizontal axes in each of the following "Parameter Sensitivity Results" figures (Figs. S2-2, S2-4, and S2-6) corresponds to this variation in control proportion (ρ).

For each variable regulated (z_B, Δz_B, w), we defined the 'baseline' value for additive noise (σ'_a) for that variable as the group mean of the within-subject standard deviation for that variable, based on the experimental data (Fig. 2):

	Position (z_B)	Heading (Δz_B)	Step Width (w)
Baseline Additive Noise (σ'_a):	0.0424 m	0.0160 m	0.0257 m

We took (as before, see Supplement S1) the baseline amplitude of multiplicative noise (σ'_m) to be 10% of the baseline additive noise (i.e., $\sigma'_m = 0.10 \cdot \sigma'_a$) and the baseline ratio of the cost function weights to be $(\gamma/\alpha)' = 0.10$.

Across the range of ρ values, we tested each model operating with its 'baseline' set of parameters and with two variations each of additive noise (σ_a) and ratio of the cost function weights (γ/α), as follows:

	Baseline Models:	To Vary σ_a:	To Vary γ/α:
σ_a:	Fixed: σ'_a	Vary: $\{(½\cdot\sigma'_a)$ & $(2\cdot\sigma'_a)\}$	Fixed: σ'_a
σ_m:	Fixed: $\sigma'_m = 0.10\cdot\sigma'_a$	Fixed: $0.10\cdot\sigma'_a$	Fixed: $0.10\cdot\sigma'_a$
γ/α:	Fixed: 0.10	Fixed: 0.10	Vary: {1 & 2}
Plot Color:	Black	Blue & Cyan	Red & Green

We also took (as before, see Supplement S1) the 'desired' or 'goal' values for each variable to be:

Position: $z^*_B = 0.0$ m<br>(center of path)	Heading: $\Delta z^*_B = 0.0$ m<br>(straight ahead)	Step Width: $(w^*) = 0.127$ m<br>(from experiment – Fig. 2)

For each model and each set of parameter values tested, we generated 20 sets of time series data, each simulating 500 consecutive walking steps (analogous to simulating 20 walking trials). Time series of left & right foot positions (z_L and z_R), body position (z_B), heading (Δz_B), and step width (w) were generated for each simulated walking trial and analyzed in the same manner as experimental data: standard deviations (σ) and DFA exponents (α) were computed for each time series.

We also computed (as before, see Supplement S1) the percentage of steps (z_L and z_R) taken in each simulated trial that exceeded the *Lateral Boundary Limits* (±0.885 m) or the Step Width Limits (±5σ).

Figures and Plotting Conventions:

For each multi-objective control model, we present two figures. In the first figure for each model (odd numbered figures), we display example time series and comparisons to human values for 'baseline' parameter values. For each output variable, we show comparisons of boxplots to demonstrate the range and distribution of values within each data set.

For each second (even numbered) figure, we show the mean ± 1σ bands for humans (gray shaded areas) to indicate the *range* of values observed experimentally for that variable. We plot simulation results as mean ±95% confidence intervals for each mean to identify where the predicted simulation means would lie inside or outside of the experimental range.

Heading & Position Control:

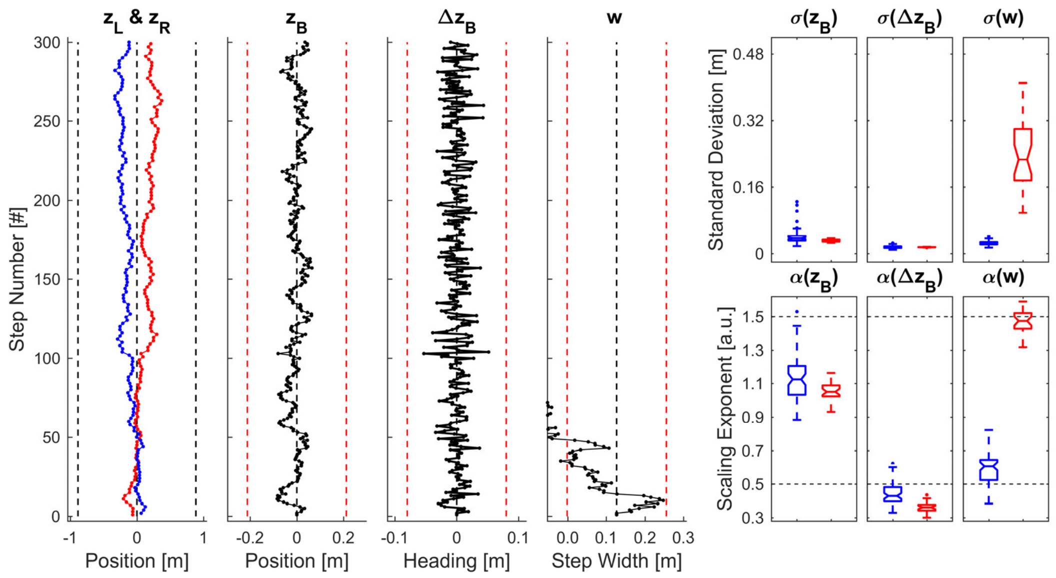

Figure S2-1: Example Results. Left: Typical time series of left & right foot placements (z_L & z_R), body position (z_B), heading (Δz_B), and step width (w) for baseline parameter values (see text). Right: Box plots of variability (standard deviations) and DFA α results for each variable for the baseline model (red: 30 simulated trials of 500 steps each) and humans (blue: 65 total trials of 290 steps each from 13 participants (5 trials each)) . Results shown for proportion ρ = 0.15 (85% Δz_B / 15% z_B).

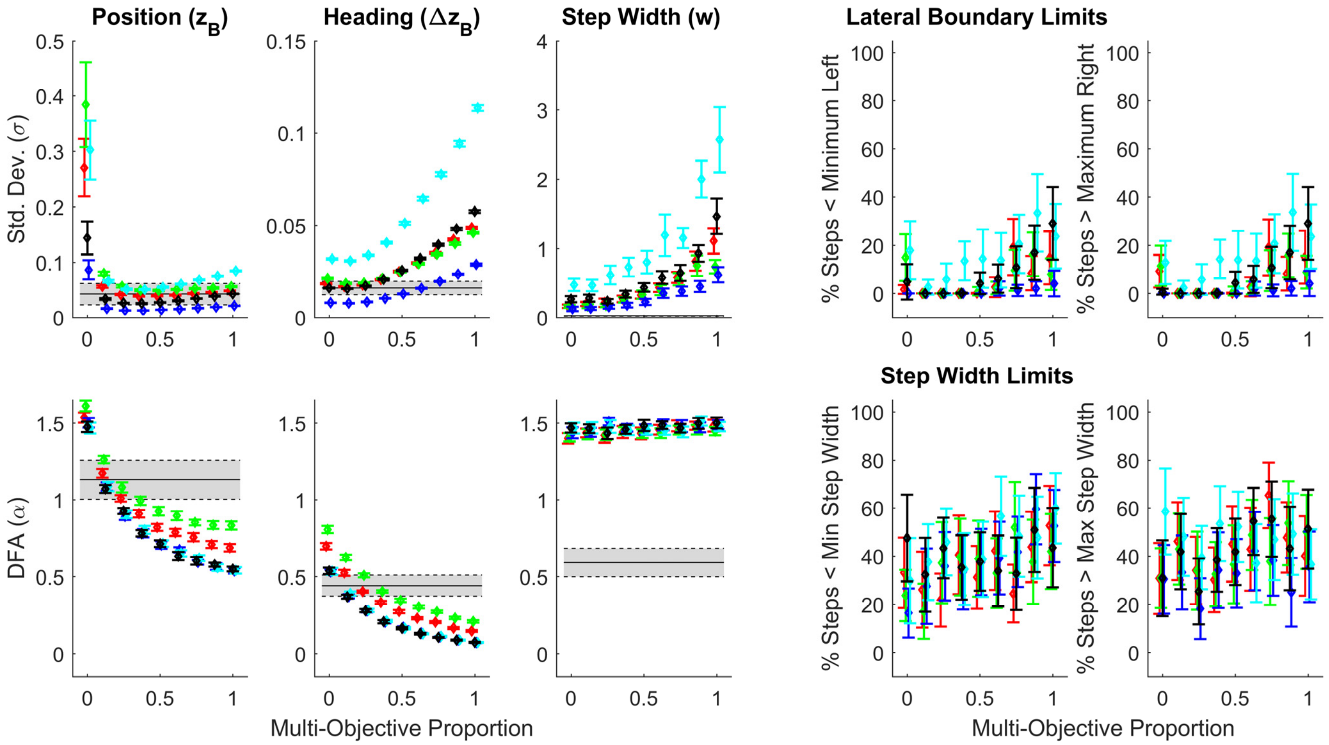

Figure S2-2: Parameter Sensitivity Results. Each set of point represents variations across proportion (ρ) for baseline parameter values (Black) and for variations in additive noise (σ_A: Blue & Cyan) or relative cost function gains (γ/α: Red & Green) (see text). Left: Variability and DFA α results. Error bars indicate ±95%CI for the mean of each set of simulations. Gray horizontal bands indicate the range (mean±SD) exhibited by humans in the experiment. Right: Percentage of steps across trials that exceeded either lateral boundary or step width limits. Error bars indicate ±95%CI for each mean.

Heading & Step Width Control:

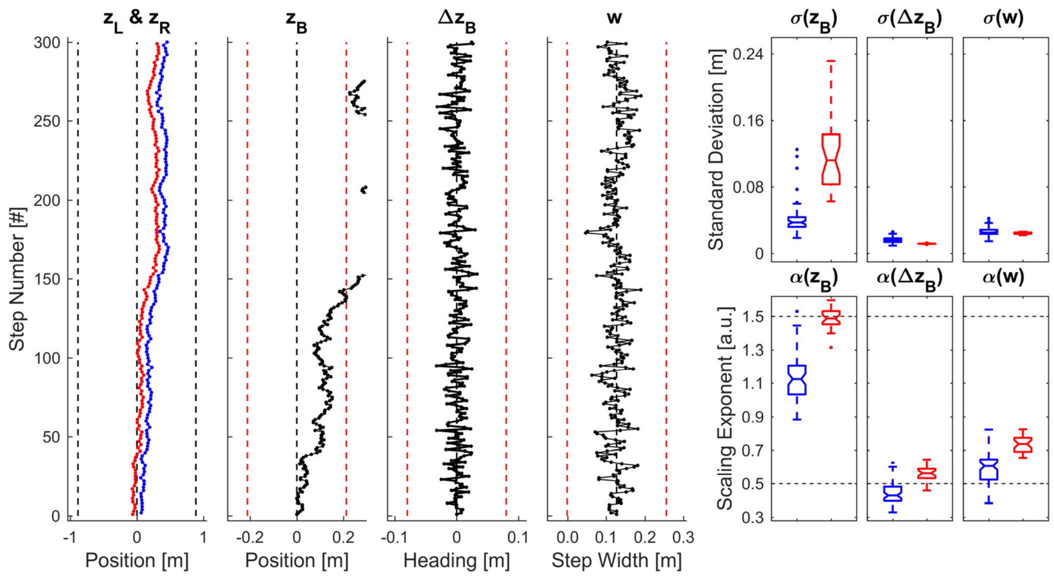

Figure S2-3: Example Results. Left: Typical time series of left & right foot placements (z_L & z_R), body position (z_B), heading (Δz_B), and step width (w) for baseline parameter values (see text). Right: Box plots of variability (standard deviations) and DFA α results for each variable for the baseline model (red: 30 simulated trials of 500 steps each) and humans (blue: 65 total trials of 290 steps each from 13 participants (5 trials each)) . Results shown for proportion ρ = 0.50 (50% Δz_B / 50% w).

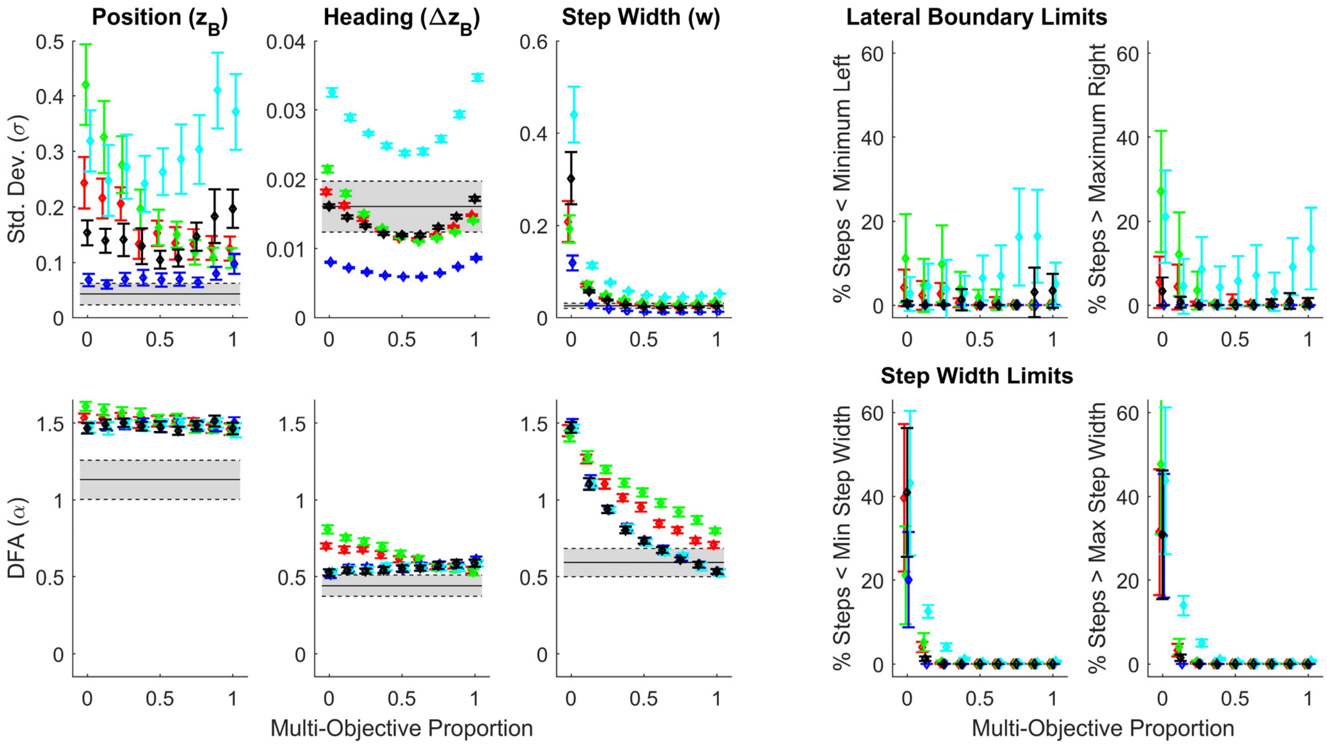

Figure S2-4: Parameter Sensitivity Results. Each set of point represents variations across proportion (ρ) for baseline parameter values (Black) and for variations in additive noise (σ_A: Blue & Cyan) or relative cost function gains (γ/α: Red & Green) (see text). Left: Variability and DFA α results. Error bars indicate ±95%CI for the mean of each set of simulations. Gray horizontal bands indicate the range (mean±SD) exhibited by humans in the experiment. Right: Percentage of steps across trials that exceeded either lateral boundary or step width limits. Error bars indicate ±95%CI for each mean.

Position & Step Width Control:

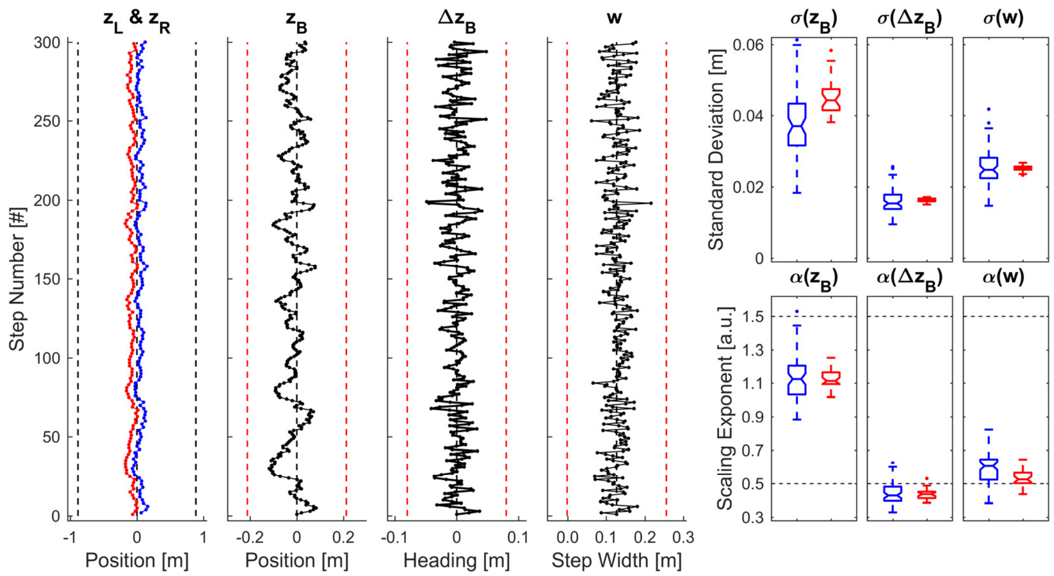

Figure S2-5: Example Results. Left: Typical time series of left & right foot placements (z_L & z_R), body position (z_B), heading (Δz_B), and step width (w) for baseline parameter values (see text). Right: Box plots of variability (standard deviations) and DFA α results for each variable for the baseline model (red: 30 simulated trials of 500 steps each) and humans (blue: 65 total trials of 290 steps each from 13 participants (5 trials each)) . Results shown for proportion $\rho = 0.93$ (7% z_B / 93% w).

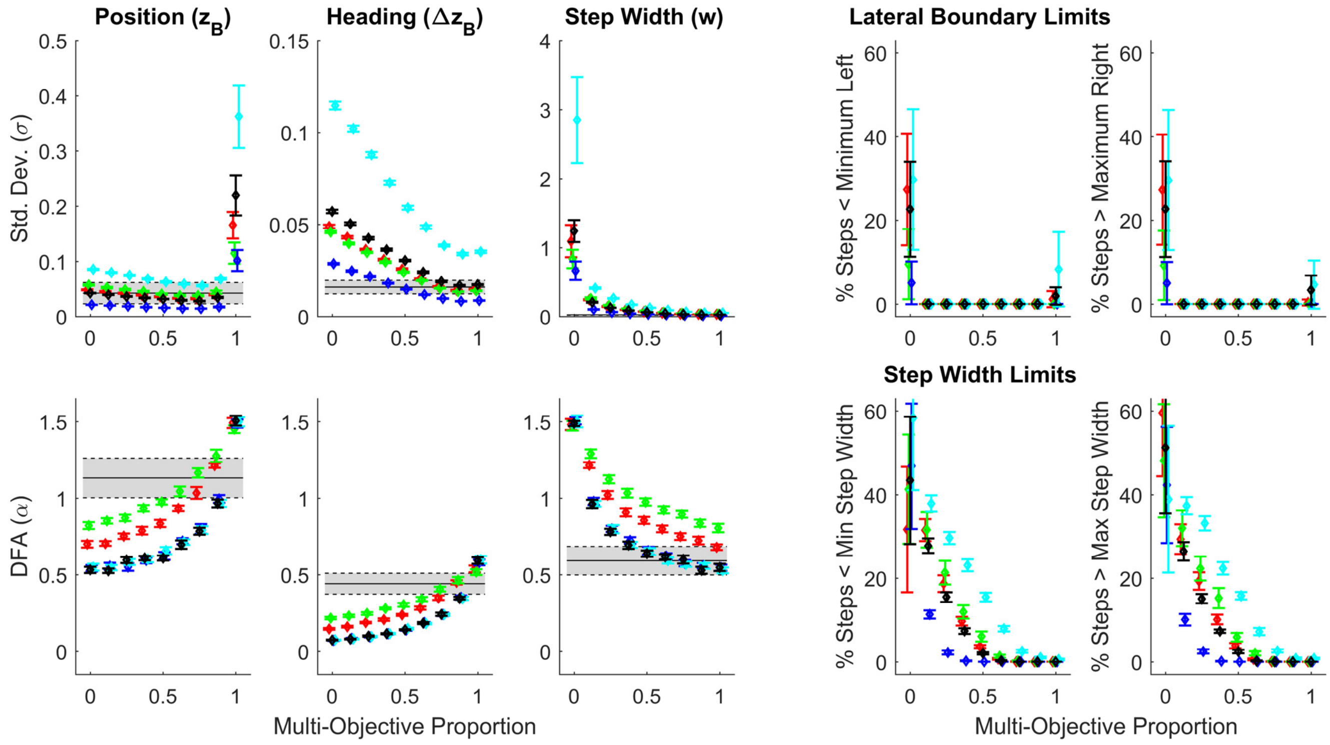

Figure S2-6: Parameter Sensitivity Results. Each set of point represents variations across proportion (ρ) for baseline parameter values (Black) and for variations in additive noise (σ_A: Blue & Cyan) or relative cost function gains (γ/α: Red & Green) (see text). Left: Variability and DFA α results. Error bars indicate ±95%CI for the mean of each set of simulations. Gray horizontal bands indicate the range (mean±SD) exhibited by humans in the experiment. Right: Percentage of steps across trials that exceeded either lateral boundary or step width limits. Error bars indicate ±95%CI for each mean.

NOTE: See also Fig. 6 in main paper for a closer view of parameter sensitivity results over the range $\rho \in \{0.89, \ldots, 0.97\}$.

Summary / Conclusions:

Multi-Objective Control of Heading and Position or Heading and Step Width (Figs. S2-1 to S1-4):

All of these models failed to capture the stepping dynamics exhibited by humans (Fig. 2) across all parameter combinations tested. In addition to not matching variability and/or DFA results, most simulations regularly exceeded either lateral boundary or step width limits, or both. In addition, control of Δz_B and z_B (Fig. S2-2) always resulted in $\alpha(w) \approx 1.5$, while control of Δz_B and w (Fig. S1-6) always resulted in $\alpha(z_B) \approx 1.5$. In each case, the DFA $\alpha \approx 1.5$ for the variable that was not regulated reflected an uncontrolled random walk process [1, 2]. Thus, similar to the uni-objective models (see Supplement S1), the failure of either of these multi-objective models to replicate human stepping dynamics was not due to some limitation in the choice of parameters, but rather to the fundamental structural of these models themselves.

Adding Biomechanically Relevant Constraints: Given than none of the above models replicated human stepping dynamics, we could have next run new simulations while adding the same biomechanically relevant constraints to foot placement that we did for the uni-objective control models (see Supplement S1). As in the uni-objective case, adding such constraints would have guaranteed that these multi-objective models could successfully achieve the walking task (Eq. 1) and would do so while only taking steps that were biomechanically feasible. However, we did not run such models because, based on the results of our constrained uni-objective models (Supplement S1; Figs. S1-7 thru S1-12), it was clear none of these simulations would have replicated human stepping dynamics (Fig. 2). Adding constraints to the heading – position control model would only result in that model regularly hitting the step width constraints, similar to Figs. S1-7 & S1-9. Adding constraints to the heading – step width control model would still result in excessive lateral motion of that model, similar to Figs. S1-9 and S1-11. Neither would have replicated the stepping dynamics we see in humans (Fig. 2).

Multi-Objective Control of Position and Step Width (Figs. S2-5 and S2-6):

For most values of control proportion (ρ), this model also failed to capture the stepping dynamics exhibited by humans (Fig. 2). Model outputs did not match the variability and/or DFA results exhibited by humans, and many of these simulations regularly exceeded either lateral boundary or step width limits, or both.

However, over a relatively narrow range of ρ values that reflected control that was weighted "mostly" to step width, but "slightly" to lateral position (i.e., $\rho \in \{\sim 0.85, \ldots, \sim 0.98\}$) and for a relatively narrow range of parameter variations around the 'baseline' set of values (black symbols in Fig. S2-6), this model successfully accomplished the task (i.e., satisfied Eq. 1) without stepping off of the path and without taking biomechanically unrealistic steps, and also exhibited stepping dynamics that matched those observed by humans across all three variables (z_B, Δz_B, and w) and for both standard deviations and DFA a exponents of each of these variables. (See also Fig. 6 in main paper).

References:

1. John J, Dingwell JB, Cusumano JP. Error Correction and the Structure of Inter-Trial Fluctuations in a Redundant Movement Task. PLoS Comput Biol. 2016;12(9):e1005118. doi: http://dx.doi.org/10.1371/journal.pcbi.1005118.
2. Dingwell JB, Cusumano JP. Re-Interpreting Detrended Fluctuation Analyses of Stride-To-Stride Variability in Human Walking. Gait Posture. 2010;32(3):348-53. doi: http://dx.doi.org/10.1016/j.gaitpost.2010.06.004.
